# Prediction of incident myocardial infarction using machine learning applied to harmonized electronic health record data

**DOI:** 10.1186/s12911-020-01268-x

**Published:** 2020-10-02

**Authors:** Divneet Mandair, Premanand Tiwari, Steven Simon, Kathryn L. Colborn, Michael A. Rosenberg

**Affiliations:** 1 Division of Internal Medicine, University of Colorado School of Medicine, Aurora, CO USA; 2 Colorado Center for Personalized Medicine, University of Colorado School of Medicine, Aurora, CO USA; 3 Division of Cardiology and Cardiac Electrophysiology, University of Colorado School of Medicine, 12631 E. 17th Avenue, Mail Stop B130, Aurora, CO 80045 USA; 4 Department of Surgery, University of Colorado School of Medicine, Aurora, CO USA

**Keywords:** Myocardial infarction, Machine learning, Electronic health records

## Abstract

**Background:**

With cardiovascular disease increasing, substantial research has focused on the development of prediction tools. We compare deep learning and machine learning models to a baseline logistic regression using only ‘known’ risk factors in predicting incident myocardial infarction (MI) from harmonized EHR data.

**Methods:**

Large-scale case-control study with outcome of 6-month incident MI, conducted using the top 800, from an initial 52 k procedures, diagnoses, and medications within the UCHealth system, harmonized to the Observational Medical Outcomes Partnership common data model, performed on 2.27 million patients. We compared several over- and under- sampling techniques to address the imbalance in the dataset. We compared regularized logistics regression, random forest, boosted gradient machines, and shallow and deep neural networks. A baseline model for comparison was a logistic regression using a limited set of ‘known’ risk factors for MI. Hyper-parameters were identified using 10-fold cross-validation.

**Results:**

Twenty thousand Five hundred and ninety-one patients were diagnosed with MI compared with 2.25 million who did not. A deep neural network with random undersampling provided superior classification compared with other methods. However, the benefit of the deep neural network was only moderate, showing an F1 Score of 0.092 and AUC of 0.835, compared to a logistic regression model using only ‘known’ risk factors. Calibration for all models was poor despite adequate discrimination, due to overfitting from low frequency of the event of interest.

**Conclusions:**

Our study suggests that DNN may not offer substantial benefit when trained on harmonized data, compared to traditional methods using established risk factors for MI.

## Introduction

Cardiovascular disease (CVD) has long been a leading cause of death in the United States, with more than 900,000 deaths in 2016, a substantial portion of which were attributable to myocardial infarction (MI) [1]. Although there have been dramatic improvements in public health that have spurred a decline in CVD related deaths over the past several decades, incidence of CVD mortality has remained steady in recent years. As a result, considerable effort has been placed in improving risk prediction of CVD-related events [2, 3]. Despite this, clinical practice has remained largely unchanged, relying on traditional scoring metrics such as Thrombolysis in Myocardial Infarction (TIMI) Risk Score [4] for risk stratification.

Machine learning (ML) has risen as a contemporary method of prediction. ML methods offer an approach that contrasts with typical statistical tools to extract relationships between variables in a training dataset to predict various outcomes, including mortality. Numerous studies have applied these techniques to predicting cardiac events primarily in patients presenting with acute coronary syndrome (ACS), in some cases showing outperformance of traditional risk methods and statistical techniques [4–10]. A specific class of these techniques, deep learning, has received significant interest in recent years. A form of representation learning, deep learning allows a machine to learn patterns based on raw input of data without any prior variable engineering [11]. Successful applications of deep learning have been diverse, from playing poker [12] to detecting mammographic lesions [13]. Studies have demonstrated deep learning can improve upon traditional ML methods in predicting mortality, by capturing non-linear relationships among predictor variables [14] and through the incorporation of ‘unstructured’ data such as word embeddings in discharge summaries [15].

Use of Electronic Health Record (EHR) data has increasingly been the focus of such prediction efforts. EHR data offers numerous advantages such as longitudinal follow up and large, more generalized cohorts for study [16]. Applications in cardiology have included prediction of lifetime costs associated with CVD, phenotyping patients most likely to benefit from lipid pharmacotherapy, and estimating 1 year mortality risk in the ICU setting. Perhaps most exciting is the use of EHR data to facilitate the development of learning healthcare systems, where data can be shared across institutions and used to enhance models for real-time clinical prediction. Prior work has demonstrated that clinical ‘phenotypes’ can be developed that describe institutional patient populations and are stable from institution to institution [17]. Harmonization is a method of sharing these representations across institutions in a way that is translatable and preserves patient privacy [18].

Here we explore the application of ML methods to a harmonized dataset to predict incident MI at 6 months. Prior work, particularly studies that have used deep learning, have largely limited the populations studied for MI prediction to those patients that present to the ED or cath lab for ACS [14, 15, 19, 20]. And while prior studies have used EHR data for MI prediction, none to our knowledge have used harmonized datasets [5, 21–23]. We aim to test the efficacy of ML methods in this context compared to more traditional statistical approaches. Our end goal is to develop a prediction system that not only meaningfully predicts MI incidence over a clinically-relevant time horizon in an undifferentiated patient but also can be integrated with EHR systems across institutions to facilitate the advent of learning healthcare systems. Among the characteristics we examined in this developmental process includes identification of the appropriate data resampling to manage dataset imbalance, and development of a classification algorithm based on training time and accuracy.

## Methods

We conducted a systematic examination of EHR data sampled from over 2 million individuals, in whom we have harmonized 52,000 features, including diagnoses, medications, and procedures under the Observation Medical Outcomes Partnership common data model (OMOP-CDM). The code used for the analyses, as well as the model weights and mapping (OMOP-CDM input codes) for the final model, are available in the supplemental material. This investigation was approved by the University of Colorado Multiple Institutional Review Board (COMIRB), with permission for data access under the Health Data Compass honest-broker agreement. De-identified data was used for all analyses; no individual patient information was accessed in conduct of this investigation.

### Study population and case ascertainment

The UCHealth hospital system includes 3 large regional centers (North, Central, South) over the front range of Colorado that share a single Epic instance, which allows data from all centers to be pooled into a single data warehouse, a copy of which is located on the Google cloud platform. This warehouse of data was queried using Google BigQuery to create a dataset and conduct analyses directly on the Google cloud platform, where an array of machine-learning tools can be run on virtual machines. To create our study dataset, we applied a classification approach based on predicting risk of incident MI over a 6-month period. We performed a SQL query on the UCHealth EHR for subjects, first extracting patients that had MI, excluding patients who had a prior diagnosis of MI. For these patients with incident MI, the index date was 6 months prior to the MI event and all data prior to this date was used for medical history. For the cohort of control patients without MI, the index date used was 6 months prior to the last recorded encounter date in our dataset and all data prior to this date was again used for medical history. Data was gathered during the time period 2003 to 2018.

### Common data model and data splitting

We used a common data model for EHR data, based on the Observational Health Data Sciences and Informatics (OHDSI) collaboration, which uses OMOP-CDM [24–26]. The OMOP CDM is a mapping of the raw EHR data to a harmonized dataset; we used this CDM with 52 k variables (i.e., features) from the EHR, including age, sex, diagnoses, procedures, and medications. The missing data assumption for this study was that if a given feature, other than age or sex, was absent then this feature was assumed to not be present for that person. Preliminary studies identified a substantial decrease in analytical time using the top 8500 most common (across the entire EHR) concepts, which were used as input into prediction models. The final dataset was composed of 2.27 million records, which was then split into training (80%), dev for hyperparameter tuning (0.3%), and testing (19.7%) sets to compare the models developed in this investigation.

### Model development

For all models, hyperparameter tuning was performed using iterative random sampling of 10,000 records for manual grid search (neural networks), and 10-fold cross validation for automated grid search (for other machine learning approaches).

Due to the relative infrequency of the outcome (6-month incident MI) across the dataset (0.91%), there is substantial imbalance between the cases and controls in this investigation. The presence of such imbalance can produce classifiers that default to assigning new cases to the majority class, thus having very poor accuracy overall [27]. Re-sampling techniques have increasingly been studied as a potential solution to this, with both oversampling techniques - which increase frequency of rare cases relative to controls - and undersampling techniques - which reduce frequency of controls relative to cases - used. We examined several strategies for resampling, including random oversampling, synthetic minority oversampling technique (SMOTE) [27], random undersampling, and cluster centroid. To identify the best resampling approach, we used a deep neural network (DNN) (7 layers × 100 neurons/layer), as pilot analyses using a smaller dataset suggested this approach might be superior to other ML approaches. We also compared with a model using no resampling (imbalanced).

Once we identified an optimal resampling approach, we compared several classification algorithms, including naïve Bayesian classification, regularized logistic regression, random forest classification, boosted gradient classification, one-layer fully connected neural networks (shallow) and multiple layer fully connected neural networks (deep). Parameters for the DNN were obtained by hyperparameter tuning using manual grid search, with including the number of layers, number of neurons, activation function (tanh, relu, sigmoid and elu), learning rate, dropout and batch-size. Tuning yieleded a final DNN size of 7 layers and 100 neurons per layer. Model comparison was based on area-under-curve and F_1_ statistic. Loss function applied in this analysis was the cross-entropy loss. Computation time includes all prior data sampling and algorithm performance. Once an optimal model and resampling approach were identified, we conducted sensitivity analysis using several alternative resampling and modeling approaches in combination to ensure that the combination (dimensionality reduction, resampling, and classification algorithm) identified was indeed optimal. Precision-recall and receiver-operator characteristic curves, as well as feature importance plots, were created for the optimal model for manual inspection. Platt re-scaling and isotonic regression was employed to better calibrate predicted probabilities to expected distributions of the observed probabilities in the data.

### Validation of developed model

The optimal model was then compared with a simple logistic regression model without regularization based on presence of known clinical predictors of MI, using diagnosis codes (ICD-10; ICD-9) to obtain inputs of diabetes (ICD 9: 250, ICD10 EO8 – E13), hypertension (I10x; 401.x), age, sex, smoking status (ICD 9305.1, ICD 10 F17.210), and fasting lipid level.

### Computation and analysis

All analyses were run on Google Cloud Platform, using 96 CPUs and 620 GB of RAM. Scripts were composed in Python (version 3) and were run on Jupyter Notebook with Tensorflow platform on the Google Cloud Platform. Machine learning packages included *scikit-learn* and *keras.* Confidence intervals were calculated using Wald method [28, 29], although almost all were within the rounding error of the estimates due to the large testing sample size (N = ~ 2.27 M), and are not displayed.

## Results

Across the entire dataset of ~ 2.27 million cases, approximately 21 thousand cases of MI (Table 1) were recorded. Among this group, the prevalence of cardiovascular risk factors was, as expected, significantly greater than controls. Patients with a first-MI within 6 months were older with higher rates of coronary artery disease, diabetes, hypertension, chronic kidney disease, obesity and heart failure.

**Table 1:** UCHealth Population by MI diagnosis

	No MI	6-month Incident MI
**Number (%)**	2.25 million(%)	20,591 (%)
**Age (Mean ± SD)**	43.32 ± 22.56	70.36 ± 14.02
**Female sex (%)**	1,228,689 (54.5%)	7880 (37.7%)
**Hypertension (%)**	376,371 (16.72%)	12,314 (59.8%)
**Coronary artery disease (%)**	59,199 (2.63%)	6700 (32.53%)
**Mitral valve disease (%)**	30,251 (1.34%)	1668 (8.1%)
**Heart failure (%)**	44,999 (2%)	3038 (14.75)
**Diabetes mellitus (%)**	131,059 (5.82%)	5432 (26.38%)
**Obesity (%)**	127,575 (5.67%)	2343 (11.37%)
**Chronic kidney disease (%)**	42,759 (1.9%)	2128 (10.33%)

**Legend:** Baseline demographics and relative frequency of typical MI risk factors. Diagnoses based on presence of diagnosis code (ICD-10; ICD-9) for each. Hypertension: I10x; 401.x, Coronary artery disease: I25.1; 414.01, Mitral Valve disease: I34.2, I34.0, 394.0, 424.0, Heart failure: I50.9, 428.0, Type II Diabetes Mellitus: E11.9, 250.00, Obesity: E66.9, 278.0, Chronic kidney disease: N18.9, 585.9

We next examined various re-sampling methods to address the substantial degree of imbalance in the dataset of cases compared to controls. Using a 7-layer DNN algorithm with hyperbolic tangent activation and 20% dropout, we found that random undersampling had the overall best performance by AUC and F1 (Table 2) and shortest time for algorithm time-to-completion. All re-sampling methods, including random oversampling, SMOTE, random undersampling and undersampling with cluster centroid, substantially outperformed the DNN model with no re-sampling methods used to address class imbalance (AUC 0.51, F1 0.01).

**Table 2:** Comparison of Resampling Strategies

	F1 Score	AUC	Training time
Oversampling			
Random	0.105	0.816	22 min
SMOTE	0.109	0.786	34 min
Undersampling			
Random	0.091	0.839	2 min
Cluster centroid	0.057	0.78	78 min
None	0.01	0.51	3 min

Sampling comparison from deep learning model

**Fig. 1:**
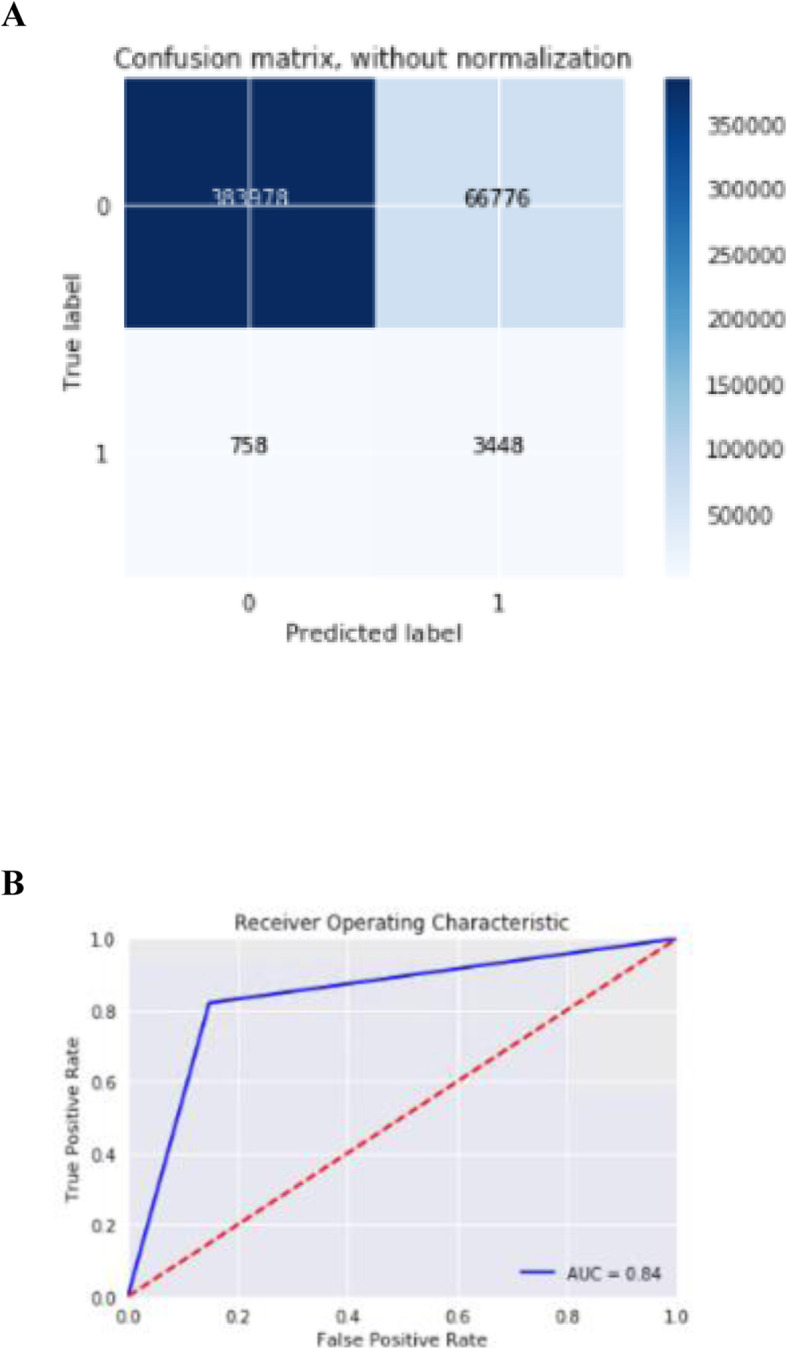
**a**. Precision-recall curve for optimal model. **b**. ROC curve for optimal model. A) Confusion matrix for the optimally performing DNN B) ROC curve for DNN model

Next, using random undersampling on the training data, we tested the accuracy of various classification models on the held-out dataset and found a DNN outperformed Naïve Bayes, logistic regression with regularization, shallow NN, random forest, and boosted gradient descent models (Table 3). Model accuracy, measured by AUC, was quite similar between the DNN and a logistic regression with regularization (AUC 0.835 vs. 0.829). The final model had a sensitivity of 0.82, specificity of 0.733, precision of 0.05 and recall of 0.82 (Fig. 1).

**Table 3:** Comparison of machine learning approaches

	F1 Score	AUC	Training time
Naïve Bayes	0.060	0.73	1 min
Logistic regression with L2 regularization	0.084	0.829	1 min
Logstic regression with no regularization	0.06	0.79	1 min
RF	0.084	0.765	3 min
Shallow NN	0.101	0.83	1 min
Deep NN	0.092	0.835	2 min
GBM	0.077	0.83	9 min

Comparison of various models using Random Undersampling technique and all features. F1 and AUC calculated from model applied to held-out testing set (20%); training time is for training of training set (80%)

Finally, the optimal model, a DNN, was compared to a logistic regression using only the known risk factors for MI listed above. The baseline model’s AUC, 0.79, and F score 0.06, both were near that of the optimal model.

**Fig. 2:**
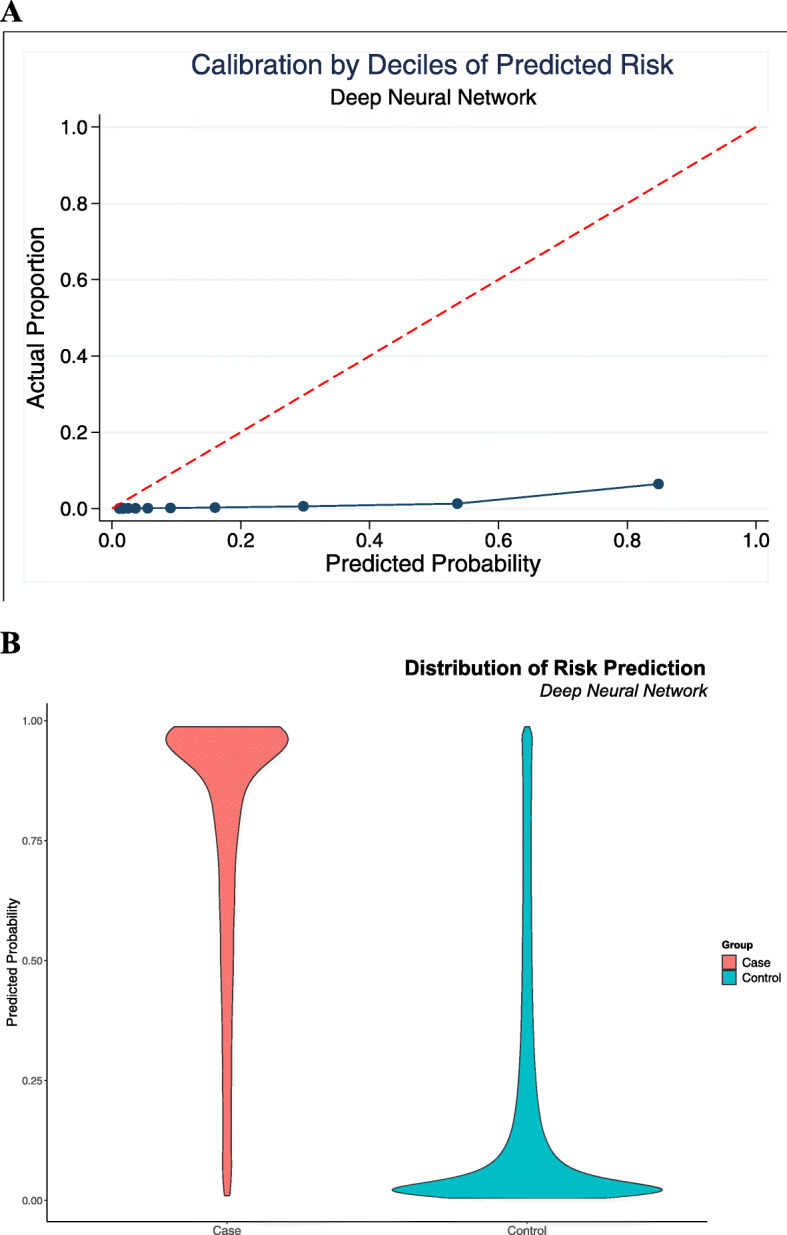
Calibration curve for optimal model. **a.** Calibration plot for the DNN, showing a wide discrepancy between actual observed distribution of outcomes vs. predicted probabilities from the model **b.** Distribution of distribution of predicted probabilities for cases vs. controls, showing good discrimination

**Fig. 3:**
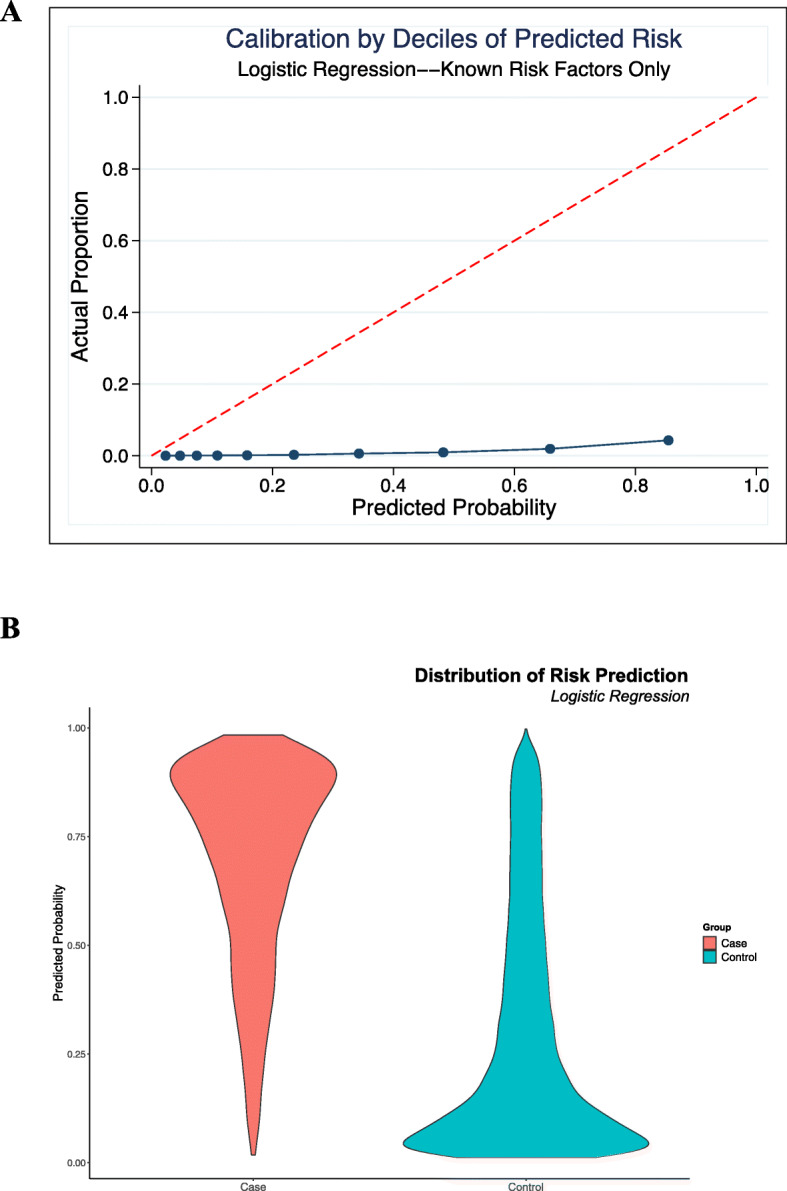
Calibration curve for comparison model. **a.** Calibration plot for the logistic model using only known risk factors, showing a similar discrepancy between actual observed distribution of outcomes vs. predicted probabilities **b.** Distribution of distribution of predicted probabilities for cases vs. controls, again with good discrimination

Calibration plots for both the optimal DNN (Fig. 2) and simple logistic models (Fig. 3) were poor, with substantial discrepancy between observed distributions and predicted probabilities. Both models, however, appear to adequately discriminate positive cases of incident MI from controls. Both Platt’s rescaling and isotonic regression were employed with no improvement in model calibration.

## Discussion

In this investigation of using harmonized EHR data for prediction of incident MI at 6 months, we found a DNN with random undersampling had the most accurate classification, although a simpler logistic regression using a more limited set of ‘known risk factors’ performed nearly as well. All models were poorly calibrated. While our study was ultimately negative a variety of useful insights emerged that would help guide future research in integrating ML methods with real-world clinical decision-making. Our study with over 2 million subjects and 52,000 features has the largest sample size and feature space for MI prediction to date [4–6, 8–10, 14, 15, 19, 20, 23]. As prior studies that have applied deep learning to MI prediction have focused on predicting recurring events or mortality risk in patients that presented to the ED or cath lab with ACS [14, 15, 19, 20], to our knowledge, this is the first investigation to attempt to use deep learning to predict ‘first-MI’ events in an otherwise undifferentiated patient population. It is well established that after an initial MI, rates of subsequent MI are markedly higher, up to 30%, compared to the general population [30]. The population of those with ‘first-time MI’ represents a different phenotype than has been assessed in prior studies, with expected lower rates of MI and overall improved survival outcomes. Despite the DNN’s performance, with an AUC in range of prior studies, the degree of improvement in accuracy was not substantial compared to a logistic regression with a handful of ‘known’ predictors of MI. A key explanation for this is likely the data input itself, harmonized EHR data. Prior studies have shown advanced ML methods and even DNN improve in performance relative to controls when the numbers of features increase specifically when using EHR data [5, 15]. These studies, however, did not use harmonized data [31]. While harmonized data offers many benefits in real world applications, such as integration immediately with healthcare systems and allowing for direct application and validation to data mapped from a separate EHR, there are substantial challenges to successfully deploying complicated ML methods to learn from this data. Numerous variables have not been harmonized across datasets via the OMOP-CDM, many of which are more granular, ‘unstructured’ data that may specifically benefit deep learning methods. Payrovnaziri et all, in fact, noted that word embeddings derived from discharge summaries were critical training inputs to improve DNN’s predictions compared to controls when using EHR data [15]. Novel biomarkers [32, 33] and features of both echo [34] and cardiac MRI [35] imaging modalities are being developed as markers of acute MI and again, represent ‘unstructured data’ not currently included in OMOP-CDM. While it is not new that a logistic model can perform similarly to ML methods in the context of MI mortality prediction [8, 10], it is striking and distinct from prior studies that a logistic model with a feature space substantially reduced to only known risk factors performed similarly to the DNN trained on the full feature set. This reflects that most of the risk prediction ‘content’ of the harmonized dataset is already captured by known risk factors for MI. Deep learning, which thrives on uncovering complex, nonlinear relationships between variables, may ultimately show improved performance with more granular data, but such granular data would limit the key benefit of harmonization in that it allows models developed at individual institutions to speak to each other in the same global space [18]. This trade-off between use of data inputs that can be replicated across populations such as harmonized data with data types that are more granular and thus likely to be more predictive, is reflective of the bias-variance trade-off in ML. Moreover, as harmonization is a crucial step towards incorporating prediction models in real-world EHR systems at scale, these limitations of advanced ML methods, specifically DNN, are a very significant consideration for practical development of risk prediction tools deployed at scale across institutions.

An interesting observation from our analysis is that predicting an outcome over a short time frame appears to be poorly calibrated regardless of which predictive approach is used, even for models having good discrimination. Despite considerable efforts to improve calibration of the final model, including Platt rescaling and isontonic regression [36, 37], the final model’s calibration, or measure of how well predicted probabilities align with observed distributions of events, was extremely poor (Fig. 2). Calibration was similarly poor for the simpler logistic regression, despite its discrimination also appearing to be adequate (Fig. 3). The pattern of these calibration plots, while having good discrimination, suggests each of the models overpredicts events at all probabilities. This is consistent with the extremely high degree of imbalance in our dataset - as our approach aimed to be a screening mechanism integrated into EHR systems, the size of the dataset was substantially larger than any prior study, and as a result, the event of interest, i.e., a ‘first’ MI in 6 months, was very rare. Prior studies that have explicitly evaluated calibration when using ML methods for risk stratification [5, 38], for instance, had events of interest with 5–10 fold higher occurrence than in our study. Our prior study, another study with significant dataset imbalance in predicting a rare event, noted similar limits with accuracy and calibration of models in the context of prediction of first time occurrence of atrial fibrillation [39]. As all models in this investigation were poorly calibrated, regardless of complexity, it may be that the use of prediction methods has inherent limitations when applied as a screening tool for MI prediction in undifferentiated patients. Alternatively, it may simply be that the time horizon of 6 months, although a clinically relevant window, may be too short for such methods to show utility. In addition to highlighting these limitations, our study demonstrates the necessity of systematically evaluating model calibration. Few prior studies in MI prediction using ML have done so, despite calibrated models ultimately providing more interpretable probabilities that could be used as individual-level predictions and thus meaningfully impact clinical practice.

### Limitations and future considerations

Our study has several limitations. For one, our study included a very simple method for the temporal relationships between features in our dataset, which did not account for time-varying effects or censoring. For instance, a diagnosis or medication that was given one month before a first MI event was weighted the same as one given 4 years prior. While we suggest a 6-month time frame is reasonable for short-term prediction, we did not rigorously test this assumption and additional information about temporal risk will be needed for more accurate prediction approaches. More sophisticated methods, such as recurrent neural networks or parametric survival functions could provide more accurate prediction in future investigations. Assessing the stability of model predictions across a range of time intervals would not only help with model selection but also provide clinically relevant stratification of patients that may not have immediate but do have heightened risks for MI.

Another weakness of our study, already noted above, is that we necessarily excluded some data elements, including lab values, diagnostic tests, imaging and even reports that may have proven useful for MI prediction. In part this is due to the lack of harmonization of some variables via OMOP-CDM.

A final limitation of our study is we necessarily limited the event of interest to first-MI. As MI events were obtained using ICD codes, it was impractical for us to obtain and model repeat MI events. Repeat MI’s, however, are of substantial clinical interest as risk and likelihood for MI heighten substantially after an initial event.

## Conclusion

We studied the development of an ML model to predict 6-month occurrence of incident MI using harmonized EHR data and found that the combination of random undersampling and a DNN classification provided superior prediction than other models. However, a logistic model using a much smaller set of only ‘known’ risk factors for MI was nearly as accurate in prediction, while all final models remained poorly calibrated. ML methods, specifically DNN, may have limited benefit over more traditional MI risk prediction tools when using harmonized data until more granular data can readily be incorporated. Our methodology and use of harmonized data is an important step for developing prediction tools that can be scaled into real-world clinical practice. Future studies should also address prediction of varying time horizons, assessing for improvements in model calibration with more extended prediction intervals.

## Supplementary information

**Additional file 1 **Python code used for analysis. **Supplemental Figure 1.** Calibration curve for optimal model with results from re-scaling methods

## Data Availability

The datasets used and/or analyzed during the current study are available from the corresponding author on reasonable request.

## Abbreviations

CVD: Cardiovascular disease
TIMI: Thrombolysis in Myocardial Infarction
ML: Machine learning
ACS: Acute coronary syndrome
EHR: Electronic Health Record
OMOP-CDM: Observation Medical Outcomes Partnership common data model
SMOTE: Synthetic minority oversampling technique
DNN: Deep neural network
OHDSI: Observational Health Data Sciences and Informatics
