# Supplementary material for: Prediction of incident myocardial infarction using machine learning applied to harmonized electronic health record data
Source: BMC Med Inform Decis Mak. 2020 Oct 2;20:252. doi: 10.1186/s12911-020-01268-x (PMC7532582; doi:10.1186/s12911-020-01268-x)
Supplement: Supplementary file 1 — Additional file 1 Python code used for analysis. Supplemental Figure 1. Calibration curve for optimal model with results from re-scaling methods [file 12911_2020_1268_MOESM1_ESM.pdf]

Supplemental Figure 1. Calibration curve for optimal model with results from re-scaling methods

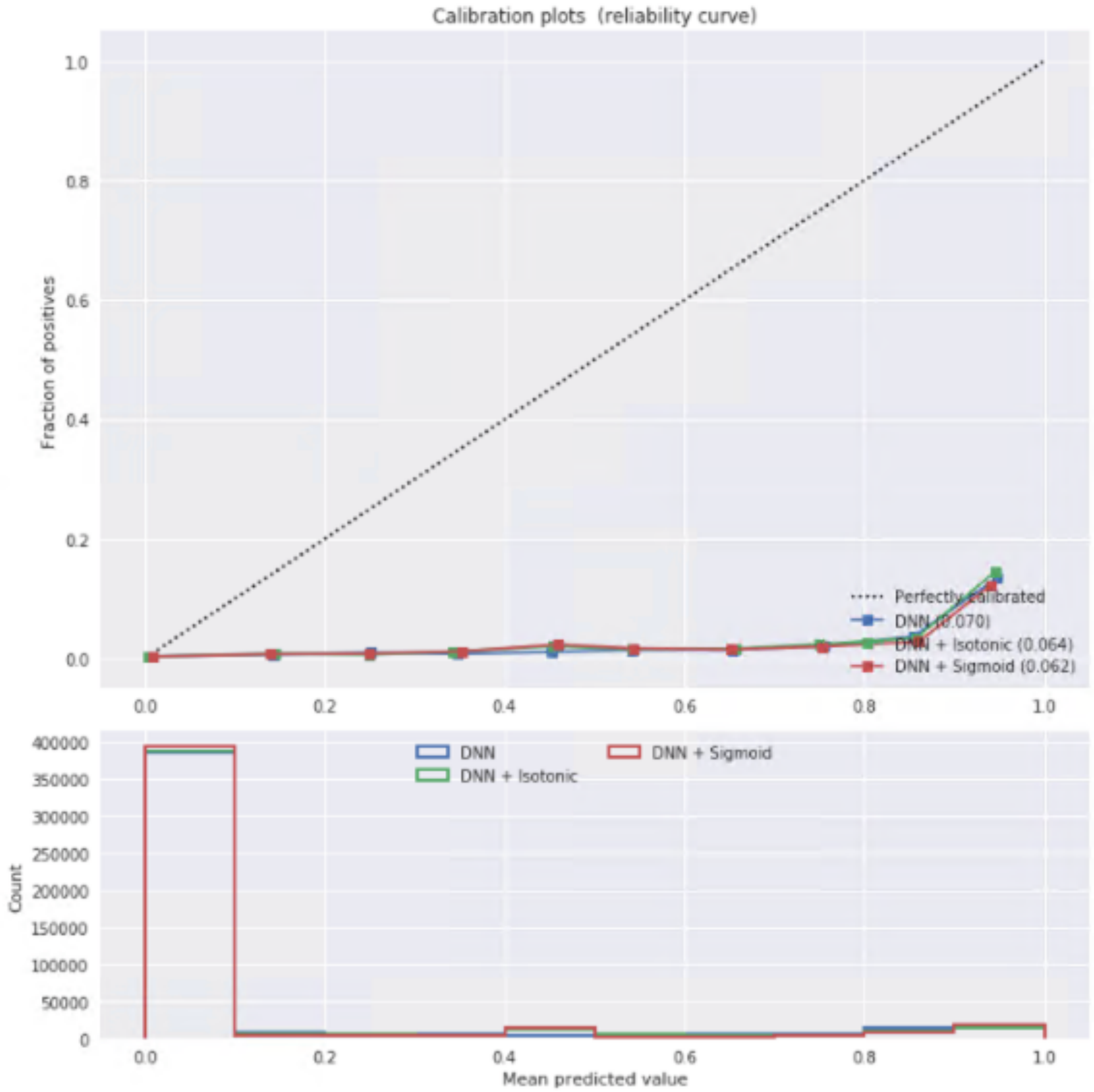

Legend: Calibration plot for the DNN with alternative curves demonstrating lack of improvement in calibration with use of rescaling methods (both Platt's rescaling and isotonic regression).

Python Code:


```
{
 "cells": [
  {
   "cell_type": "code",
   "execution_count": null,
   "metadata": {},
   "outputs": [],
   "source": [
    "!pip install keras==2.2.4\n",
    "!pip install imblearn"
   ]
  },
  {
   "cell_type": "code",
   "execution_count": null,
   "metadata": {},
   "outputs": [],
   "source": []
  },
  {
   "cell_type": "code",
   "execution_count": 2,
   "metadata": {},
   "outputs": [
    {
     "name": "stderr",
     "output_type": "stream",
     "text": [
      "/usr/local/envs/py3env/lib/python3.5/site-packages/h5py/__init__.py:36: FutureWarning: Conversion of the second argument of issubdtype from `float` to `np.floating` is deprecated. In future, it will be treated as `np.float64 == np.dtype(float).type`.\n",
      "  from ._conv import register_converters as _register_converters\n",
      "Using TensorFlow backend.\n"
     ]
    }
   ],
   "source": [
    "#from MulticoreTSNE import MulticoreTSNE as TSNE\n",
    "import pandas as pd\n",
    "import glob\n",
    "import numpy as np\n",
    "import google.datalab.bigquery as bq\n",
```

```
    "import pandas as pd\n",
    "import tensorflow as tf\n",
    "from tensorflow.contrib import lookup\n",
    "from tensorflow.python.platform import gfile\n",
    "import numpy\n",
    "import keras as kr\n",
    "from keras.preprocessing.text import Tokenizer\n",
    "from keras.models import Sequential\n",
    "from keras.layers import Dense, Activation, Dropout\n",
    "from keras.layers.advanced_activations import LeakyReLU, PReLU\n",
    "import itertools\n",
    "from sklearn.metrics import confusion_matrix\n",
    "import matplotlib.pyplot as plt\n",
    "import datetime\n",
    "import time"
   ]
  },
  {
   "cell_type": "code",
   "execution_count": 3,
   "metadata": {},
   "outputs": [],
   "source": [
    "def plot_confusion_matrix(cm, classes,\n",
    "                          normalize=False,\n",
    "                          title='Confusion matrix',\n",
    "                          cmap=plt.cm.Blues):\n",
    "    \"\"\"\n",
    "    This function prints and plots the confusion matrix.\n",
    "    Normalization can be applied by setting `normalize=True`.\n",
    "    \"\"\"\n",
    "    plt.imshow(cm, interpolation='nearest', cmap=cmap)\n",
    "    plt.title(title)\n",
    "    plt.colorbar()\n",
    "    tick_marks = np.arange(len(classes))\n",
    "    plt.xticks(tick_marks, classes, rotation=0)\n",
    "    plt.yticks(tick_marks, classes)\n",
    "\n",
    "    if normalize:\n",
    "        cm = cm.astype('float') / cm.sum(axis=1)[:, np.newaxis]\n",
    "        #print(\"Normalized confusion matrix\")\n",
    "    else:\n",
    "        1#print('Confusion matrix, without normalization')\n",
    "\n",
```

```
    "    #print(cm)\n",
    "\n",
    "    thresh = cm.max() / 2.\n",
    "    for i, j in itertools.product(range(cm.shape[0]), range(cm.shape[1])):\n",
    "        plt.text(j, i, cm[i, j],\n",
    "                 horizontalalignment=\"center\",\n",
    "                 color=\"white\" if cm[i, j] > thresh else \"black\")\n",
    "\n",
    "    plt.tight_layout()\n",
    "    plt.ylabel('True label')\n",
    "    plt.xlabel('Predicted label')"
   ]
  },
  {
   "cell_type": "code",
   "execution_count": 4,
   "metadata": {},
   "outputs": [],
   "source": [
    "%%bq query -n requests\n",
    "SELECT \n",
    "label,  age, sex, diag_med\n",
    " FROM `hdcmlmed.adhocs.v_mi`"
   ]
  },
  {
   "cell_type": "code",
   "execution_count": 5,
   "metadata": {},
   "outputs": [
    {
     "name": "stderr",
     "output_type": "stream",
     "text": [
      "/usr/local/envs/py3env/lib/python3.5/site-packages/ipykernel/__main__.py:40:
FutureWarning: reshape is deprecated and will raise in a subsequent release. Please use
.values.reshape(...) instead\n"
     ]
    },
    {
     "name": "stdout",
     "output_type": "stream",
     "text": [
      "Before OverSampling, counts of label '1': [16474]\n",
```

"Before OverSampling, counts of label '0': [1803364] \n",
"\n",
"f-score metric in the testing dataset: 0.07791068939155461%\n",
"AUC of test dataset is: 0.8305785067263767\n"
]
},
{
"name": "stderr",
"output_type": "stream",
"text": [
"/usr/local/envs/py3env/lib/python3.5/site-packages/matplotlib/font_manager.py:1320: UserWarning: findfont: Font family ['sans-serif'] not found. Falling back to DejaVu Sans\n",
" (prop.get_family(), self.defaultFamily[fontext]))\n"
]
},
{
"data": {
"image/png":
"iVBORw0KGgoAAAANSUhEUgAAAV8AAAEmCAYAAADFmJOIAAAABHNCSVQICAgIfAhkiAAAAAl
wSFlzAAALEgAACxIB0t1+/AAAADl0RVh0U29mdHdhcmUAbWF0cGxvdGxpYiB2ZXJzaW9uIDIuMS
4yLCBodHRwOi8vbWF0cGxvdGxpYi5vcmcvNQv5yAAAIABJREFUeJzt3Xu8VXP+x/HXPud0OYRK0u
WgkA+VqSaqn7uYJEzNIIZRyHXcxmVoDDLj8suYGWPGnaKMHzW5hUwSiXErCYWPinBISfR5XRO+
/fH+p5s2Wefs3P2WXX2++mxHmfv7/qutb7r4N2371rruxLJZBIREalbBXE3QEQkHyl8RURioPAVEY
mBwldEJAYKXxGRCh8RURioPDNM2ZWbGZPmNlyM/v3j9jPiWb2TG22LS5mtr+ZedztkPyPySH2+
myYzOwG4CNgdWAHMBK5z95d+5H5PAs4D9nH38nH38h/d0E2cmSZ3cXTZYAXX8H
rge2B3YEbgP618LudwI+yIfgrQkzK4q7DZKf1PPdxJjZNsBnwCnunnnnZYwMwaATcAA0PRWOAyd19jZ
gcB/wJuAi4DKoDL3f1eM/BywGrnD3B0L5a
e6+X9huH+BmYDfgA+ACd385rJsCvAj/AVwS2n82UEb0B1IL4C/ufn2o3y
Mcdw9gFfAwcJG7l5nZVGB/YCWQBIYAC8P+/wlcCEwCRgD/cvcSM9N9sFmAYc6u4zzKwN8DZwjLtPSf
fvQ2RjqOe76fkfoDHwaIY6fwB6Ab2AbYC2RIFzq5k1c/dhRL3pMe7xNZGqImW
1JFIKHu/tWwD5Ewx8b1msOPBXqbgv8DXjKzLZNqXYCCArQEmhIFKxVaUX0O2hLFPx3A78GuhOF6
VVmtnOoW0EUoi2IdVEa2IfneHAL8BcPcDQp0u4XzHpOy/OdHfAs5IPbC7zyP6Q+sBM9sCuBe4T8ErtU3hu
+nZFlhczbDAicCf3H2Ru38J/BE4KWX92/f73f73c
nd/EHgfOCqlzr3u/oG7ryLqqXfNcMy1ROZndfEY4/m6gHjbu/4e6vhuPOB+4EDqzBOQ1
z9zWhPd/j7ncDc4DXgNZEf9iJ1CqF76bnG+DXAcc
BawwMyeMrPda9Ceyja1Tfn+RRbt+Y2ddEPfsWGfYN8KW7r
66mzt1AZ+Cf7r6mmroiWVP4bnpeZVT7r7RHf/GVEP8H2iiP8H2iUK
quPZVt+mwj25SN24na1cHdtwYuJxrTziTjhQ4za0I0vjwCuDoMq4jUKl3p3cS4+3Izu4ponLYceIbor+
GHAge7+6XAg8AVZjaNKEEiuIrqItDFmApeZ2Y7AcqILcgCY2fZAT2AyUW/zG6Ix1bo8bCxw
NdASe3Mg2ZWMr4Gvgm9ArPxv4MmX9QmBnIJtbzW4G3nD308zsLuAOvru4KVIr1PPdBLn734
b2CKEg+Bc4FHgtVrgWmE12FfweYEco25liTgDFhX2/w/cAsAC4m6tkuIRpL/U2afXwFHBnqfgVChy
Z7m6GHLiE6GLeCqJe+ZgN1l8NjDKzWDCaWX+gL9FQC0T/Hn5qifWotF0K1mIiKxUM9XR
CQGCl8RkRgofEVEYqDwFRGJwSZ1q1lxt3Pz4urf9H9fzl7HXh93M+rEi49cT6kSntk2Y/dk3cTejTu
zVfpvq7qOusWz/n1/15i21duy4qecbg067tqm+kmxWihsWxt0E2cxsUj1fEckzifzt/yl8RSQ+iXozipA1

ha+IxEc9XxGRGBTk71i5wldE4qNhBxGRGGjYQUQkBur5iojEQD1fEZEY1HLP18waA1OBRkT5Ns7dh
5nZfUTzUS8PVU9295lmliCaPL8f0eutTnb3GWFfg/nuxbTXuvuoUN4duA8oJnqRwAXungxvPBkDtA
PmAwPdfWlVbc3fP3ZEJH6JguyW6q0Bert7F6KXtPY1s15h3e/cvWtYKt/CfTjQlSxnEL2WqvKN3MOI
3uTSAxhmZs3CNreHupXb9Q3lQ4HJ7t6B6O0vQzM1VOErIvFJJLJbquHuSXevnGSjQVgyzR/RHxgdtn
sVaGpmrYHDgEnuviT0XicRBXlrYGt3f8Xdk8BovnvfYn9gVPg8iszvYVT4ikiMar/ni5kVmtlMYBFRgL4
WVl1nZm+b2U1m1iiUtSV6TVel0lCWqbw0TTnA9u6+ACD8bJmpnQpfEYlPDsLX3SvcvStQAvQws85
EL4bdHdgbaA5cVtmCNLtIbkR51hS+IhKfwsLsliy4+zJgCtDX3ReEoYU1wL1E47gQ9Vx3SNmshOiFsZ
nKS9KUAywMwxKEn4sytU/hKyLxqeUxXzPbzsyahs/FwKHA+ymhmCAai50VNhkPDDKzRLgwtzwM
GUwE+phZs3ChrQ8wMaxbYWa9wr4GAY+n7Gtw+Dw4pTwt3WomIvGp/ft8WwOjzKyQqHM51t2
fNLPnzGw7omGDmcBZof4EotvM5hLdanYKgLsvMbNrgGmh3p/cfUn4fDbf3Wr2dFgAhgNjzWwI8
AlwbKaGKnxFJD61fJ+vu78NdEtT3ruK+kngnCrWjQRGpimfDnROU/4VcEhN26rwFZH46Ak3EZEYa
G4HEZEYqOcrIhID9XxFRGKgnq+ISAzU8xURiYF6viIiMdALNEVEYqCer4hIDDTmKyISA/V8RURioJ6vi
EjdSyh8RUTqnsJXRCQO+Zu9Cl8RiY96viIiMVD4iojEoKBAt5qJiNS9/O34KnxFJD4adhARiYHCV0QkB
gpfEZEY1Hb4mlljYCrQiCjfxrn7MDNrDzwENAdmACe5e5mZNQJGA92Br4Dj3H1+2NfvgSFABXC+u0
8M5X2Bm4FC4B53Hx7K0x6jqrbm76VGEYlfIsulemuA3u7eBegK9DWzXsANwE3u3gFYShSqhJ9L3X
1X4KZQDzPrCBwPdAL6AreZWaGZFQK3AocDHYfbpkOEZaCl8RiU0ikchqqY67J939m/C1QViSQG
9gXCgfBQwIn/uH74T1h5hZIpQ/5O5r3P0jYC7QIyxz3f3D0Kt9COgftqnqGGkpfEUkNrUdvgChhzoT
WARMAuYBy9y9PFQpBdqGz22BTwHC+uXAtqnlG2xTVfm2GY6RlsZ8RSQ2ubjg5u4VQFczawo8Cu
yRplqysglVrKuqPF2HNVP9KqnnKyKxSRQkslqy4e7LgClAL6CpmVV2NkuAz8PnUmAHgLB+G2BJavk
G21RVvjjDMdJS+IpIbGp72MHMtgs9XsysGDgUeA94HjgmVBsMPB4+jw/fCeufc/dkKD/ezBqFuxg6
AK8D04AOZtbezBoSXZQbH7ap6hhpKXxFJDY5GPNtDTxvZm8TBeUkd38SuAy4yMzMzmEo3Pjgj1RwD
bhvKLgKEA7j4bGAu8C/wHOMfdK8KY7rnARKJQHxvqkuEYaWnMV0RiU9tjvu7+NtAtTfmHRHcqbFi
+Gji2in1dB1yXpnwCMKGmx6iKwldE4pO/D7gpfEUkPnq8WGqkUcMinh3xWxo2LKKosJBHn32Ta+
+l/vZx9TlH8cufdaOiYh13j3uR2x58gaZbFXPn1b+mfUkL1pSt5cyrH+DdeQvW76+gIMF/H7iUzxct5+
gL7gDg2RG/pcmWjQFo2Xwrps+az8CL7ubCQYdwXL+9ASgqLGD39q3YofdQln69so5/C/XX/424lcf
G3k8ikWDX3Tpy1Y238viY0Tx47+2UfvwRk6bPo2nzbQF4YdJT3PG360gUFFBUWMRdt/+Txm33BO
CLzz7l2t+fz8IFn5FIJPj7yLG0KdmJZDLJ7X+9lskTHqOgsJCjTzyV408+K85Tjp3CV2pkTVk5fc/4B9+uKq
OoqIDnRl7EM/99F2vfipJWTenyi2tIJpNs16wJAJcOOYy3vJTjLr6b3dptz9+HDqTfWf9cv79zTzgY/2gh
W4WwBTh0yN/Xf37wL6fxxJS3Abhp9GRuGj0ZgH4HdOa8Ew9W8NaiRV98zphRdzLmmddo3LiY35
97Ms888TBduvdkv96Hcdavjvxe/b33OZADDu1HIpFgznuzOO20Ifzr6dcAGHbJWZz6m0vouf/BrPz2
m/UThj8x7gEWLijl389zeCggCWLv6zz89zU5HP46m6HLH27Kpono0FRlUVFhSSTSc44dj+uv+tpks
nonuovl0ZPN+6+cyumvO4AfDB/ITu1aU7L5lsB0LZlU/ru14l7H3057XGabNGIA/fejSeef/sH6wb23Y
ux/3mj1s8t35VXVLBm9WrKy8tZvWoV223fGuvUhTYlO/2g7hZbNlkfHKtWrVz/+cM571NRXkHP/Q
9eX69x8RYAPPzASE4777L1Ydy8xXZ1cVqbttqf22GzofDNUkFBglcfGsonk4fz3KvvM23Wx7Qv2Y5j+
nTnpQcu5bFbzmaXHaP/qd754DP6H9IVgL067cSOOrZvTdvumANz4u6P5w82PsW5d+odgft67C1Ne
d1Z8u/p75cWNG/Czffbgsckzc3iW+adlqzb8b8+rRzOWq/zhey9hyq63ptX/vjNs8P/EJjjl0by4cMpCRl0
cC8MlHc9lq62343Vm/5sQj9+fm/72SiooKAD775CMmPfUgyffDQv5+e1qcvF48Wbi5y
Gr5n1NTM3s7lmNjSXx6or69Yl6XX8cHY97Ar26rwTHXdpTaOGRawpW8t+J/6Zex95mTuHnQjAX+6
dRNOttuDVh4Zy9vEH8paXUl6xDoBFS1bw5nufVnmcgX27p+3dHnHAnrwy80OWrwy80MNOdSyr5cvY+qzE3
j8hbd4+pX3Wb3qWyY8NibjNgcfdhTjnp3GjXc+wJVXXglARXkFb057hQ6vHQsuv5ZRjz3PZ5/M58hDwBQ
VlZGw0aNGD1+CgOOG8Q1l52b8/Pa1Cl8c6Caqdc2e8u/WcXU6XPos09HPlu4lEefjEefjXqijz/3Fp07RP
NprPh2NWde/S96HT+cIVeOpkDqKjxwT95/6o+MHn4KB+29/HbR+38232ZK9OrXj6R
dn/eC4xx7WnX9ryKHWvf7fKbQp2Ylm27agqEEDDj7sKN5+4/UabfvTHvsyb948li35ipat22CdO9M2ZiA2+29
3YUFRVxUJ8jeH92NHTUslUbevf9ORAF95z3Z2fabV4oKCjIaqlPcnk2aadey+Hxcq5FsyZs06X3gFFHbDXZ0Zs9Z9tbCahQX9H5hjz3BAsTZdWg

GtC7p+HzF/LElLc5qMduAOzfvQNzP1kEwDZNimlQVAjAKb/Yh5dmzF0/jLBr3yvZ/YhhDBp6L1Omfc
CpV4xef5xf/qwbT784izVl5amHZ+smjdmv+67rL8JJ7WnVpoR3Zk5n9aqVJJNJpr38Au133a3K+p/O/
3D9GP/7s2ZSVlbGNs2a0/EnP2XF8mUs/WoxANNenkr7XQ2AA392BNNfngrAjNdeYsf2u+T4rDYDe
Tzmm6j8D6i2mdkxQF93Py18Pwno6e5V/l1r9tzPk512bZOT9ohUZ9iwYYwZM4aioiK6devGPffcw5
133smf//xnvvjiC1q2bEm/fv245557uOGGGxg9ejQNGjSguLiYG2+8kf322w+ASZMmcfHFF5NMJun
evTt33XUXDRs2ZNmyZZx44ol88sknNGnShDvuuIMuXbrEfNbZmf7RcvZqv02txeDOF03IKoA+/Fu/e
hPBuQzfY4HDNgjfHu5+XlXbFHc7NzeN2cSsevMWirvlx3jfi4/84OnMemmv9tsw/aPlcTejTtRm+O5y
8dNZ/T8/76+H15vwzeV9vlVNvSYiAkA9u4aWlVyG7/qp14DPiKZeOyGHxxORzUx9u4MhGzm74Fb
N1GsiIiQS2S31SU4fL65q6jUREcjvnq/mdhCR2ORx9ip8RSQ+BVm+l60+UfiKSGwUviIiMdCwg4hIDH
TBTUQkBgpfEZEY5HH2KnxFJD7q+YqIxKC2s9fMdgBGA62AdcBd7n6zmV0NnA5vvjjv8vAQGGb2e2
AIUAGc7+4TQ3lf4GagCxe3Cxu+8B9ALOSXmJw03u3jUslcHbkWjemU5AX+A2Myus5mUQN4R9dQCWEgU34edSd98VuCn
Uq5LCV0RiU9tzO7j7AnefET6vIJpXpm2GTfoDD7n7Gnf/CJhL9CKItC+DMLME0BsYF7YfBQxI2deo8
HkccEion5bCV0Rik8t3uJlZO6Ab8FooOtfM3jazkWbWLJS1BVJfplgayqoq3xZYFiYOSy3/3r7C+uWhfl
oKXxGJTUFBIqulpsysCfAw8Ft3/xq4HdgF6AosAP4aaqqbbaXIjyjPtKy1dcBOR2OTiZgcza0AUvA+4+y
MA7r4wZf3dwJPha6aXPqQrXww0NbOi0LtNrV+5r1IzKwK2AZZU1U71fEUkNrU97BDGWEcA77n
31LKW6dU+wVQ+Wrw8cDxZtYo3MXQAXidlJdBmFlDRm4Dooty4909CTwPHBO2Hww8nrKvweWzMcBz
oX5a6vmKSGxy0PPdFzgJeMfMZoayy4ATzNYgJeMfMZoayy4nuVuhKNAwwHzgTwN1nm9lY4F2iOyXOcfcKADOrfBlEITAy
5WUQlwEPmdm1wJtEYU/4eb+ZzSXq8Q8q8R6fqaEKXxGJTW0/ZOHuL5F+7LXKLlzq4+3XAD970WtXLINz
9Q6K7ITYsXw0cW9O2KnxFJDZ5/ICbwldE4qPHi0VEYqDwFRGJQR5nr8JXROKjnq+ISAzyOHsVvyvilSH
71AU0QkBgV53PVV+IpIbPI4exW+IhIfXXBLw8zOyLShu99V+80RkXyQuviLyo6j
nm4a7n1SXDRGR/JPH2Vv9mK+ZNSaqm4ALD/UZDL124GtgL3C98+
Bq3PVIBHJHwWJ7Jb6pCh29XdLwHKYP0bQQtz2ioRyQu5flHmpq4mt5qtSf1iZo1IP1mxiEhWCutb
dzYLNen5vmRmlwKNZGw/ovfXP5XbZolIPkgkslvqvqk5qE7+VAMbAK+AfwFnBVLhslIvlBww4ZuHsZ8
MewiIjUmnqWp1mpya1mWwJ/AHoTPVzxHHC9u3+b47aGTYYSTQFrgUGAq0Bu7NZa
NEJD8kslzqk5rc7dDZ3TulfH/BzGZXWVtEpIZqexzXzHYARgOtgHXAXe5+s5k1B8YA7YD5wEB3X2pm
CeBmoB+wEjjZ3WeEfQ0Grgi7vbdR4Xy7sB9RNfCJgAXuHuyqmNU1daa9Hw/DjutPLnmwIc12E5E
JKMcPGRRDlzs7nsAvYBzzKwj0d/aJ7t7B2By+A5wONAhLGcQPVRWmXPDgJ5AD2CYmTUL29we6lZ
u1zeUV3WMtDLNanZ9+LgceMvMKh8nPgp4tppfgIhItWq75+vuC4AF4fMKM3uPaNC5b3wQ45Z8Fa6LK
mEI0bUJ/YLS7J4FXzaypmbUOdSe4+xYDCeznCMtDL1fCvCMpeoi7
0kLKOAz6r/NYiIZJbL+3zNrB3QDXgN2D4Ec2VAtwzV2gKfpmxWGsoylZemKSfDMdLKNKvZlZk2FBH
5sXL1hJuZNQEeBn7r7l9H84Gla4ByY0oz1qN3mRhZr2BrkDjyjJ3v77qLUREqpeLByfMrAFR8D7g7o
+E4oVm1trdF4RhhUWhvBTYIWXzEqLJw0r5bgihsnxKKC9JUz/TMdKq9oKbmV1LNPB8GbAL8Ftgz+
q2ExGpTm3fahbuXhBvOfuf0tZNR4YHD4PBh5PKR9kzE4E+ZtYsXGjrA0wM61ab2Y
Wa9wrEEb7CvdMdKqSc93ANG4yXR3HxJu5fhHDbYTEckoBw9Z7AucBLxjZjND2eXAcGCsmQ0BPPg
GODesmEN1mNpfoVrNTANx9iZldA0wL9f5UefENOJvvbjV7OixkOEZaNQnf1e6+1swwsyJ3/9TMdq
zBdiIiGdV29rr7S1TdST4Tf0kcE4V+xpJ9DZhuXTc5pyr9Kd4yq1CR8V5hZMfAKcK+ZfU5087KIyI9S
3ybLyUZNHrI4kShsLwbmAY2opjstIlIT+TylZE1mNau8+krcGvT5IRGpRPk+sk+kJtwfJcP+au5+QkxaJSN
7I4+zN2POt80eIl067pa4PGZt8OddkcqPuP98sdSrZOu4mbHbyecw30xNuI+qyISKSf2py0am+qtETb
iIiuZDPL9BU+IpIbPI4exW+IhKffB7zrdGQi5kdaGZnzhc8tzWyX3DZLRPJBDiZT32ZLRPJBDiZT32zUZGKdS4DriR6ygG
hms/ty2CYRyRP5/JBFTXq+JxFNrfYNgLt/AjTNYZtEjE8UJBJZLfVJTcJ3lbuv3aBMczuIyI9WkOVSn9Tk
gltpmOcyGeavvAx4L7fNEpF8UM86s1mpSfieD/yLaAq1lcCrwPG5bJSI5If6NpSQjZpOrNPbzLYCLCtx9
ee6bJSL5II+zt/rwNbM+G3wHwvNf5fr1E6yUJNhh9S3GDcmen/bTEDhKyI/inq+Gbj7
/qnfzWxP4LyctUhE8kYed3yzv3vD3d8BuJ3u/lOfZDvmWwDsXZPtRESqk88932zHfUMuJ3u

M2MDfNEZF8ovCtgpkVANe5+3/qqD0ikkfyeVazjOHr7uvM7CpA4Ssita62e75mNhI4Eljk7p1D2dXA
6cCXodrl7j4hrPs9MASoAM5394mhvC9wM1AI3OPuw0N5e+AhoDkwAzjJ3cvMrBEwGugOfAUc5+
7zM7W1JhfcZphZ95qduohIzeVgVrP7gL5pym9y965hqQzejkRP63YK29xmZoVmVgjcChwOdAR+Fe
oC3BD21QFYShTchJ9L3X1X4KZQL6OahG8v4FUze9vMXq5carCdiEhGtT2rmbtPBZbU8PD9gYfcfY2
7fwTMBXqEZa67f+juZUQ93f5hbpvewLiw/ShgQMq+RoXP44BDQv0q1eSC22U1PBERkawU1t1UZ
eea2SBgOnCxuy8F2hLNVVOpNJQBfLpBeU9gW2CZu5enqd+2cht3Lzez5aH+4qoaVGX4mtkIdx/i7p
NreHIiIlkpqJt7d28HrgGS4edfgVMh7cGTpB8RSGaoTzXr0srU8+2WaUMRkR+rLm52cPeFlZ/N7G7g
yfC1FNghpWoJ8Hn4nK58MdDUzIpC7ze1fuW+Ss2sCNiGaoY/6tv8xCKyGamLd7iZWeuUr78AZoXP
44HjzaxRuIuhA/A6MA3oYGbtzawh0UW58e6eBJ4HjgnbDwYeT9nX4PD5GOC5UL9KmXq+e5rZojT
lCSDp7i0z7VhEpDq1PZ+vmT1I9NqzFmZWCgwDDjKzrkTDAPOBMwHcfbaZjQXeJXqA7Bx3rwj7OR
eYSHSr2Uh3nx0OcRnwkJldC7wJjAjlI4D7zWwuUY+32jnPE8lk+nA2s9lAv6o2dPePq9t5tlaXZx4jqS8
aF8Hq8urr1QdV/fdV3xQ3SLBqbd6ca60l5t2vfZzVL+30njvVm6cyMvV81+QiYEVEKulNFumV1VkrR
CQv5XH2Vh2+7t6rLhsiIvknn6/4a2pIEYmNJtYREYlB/kavwldEYlSonq+ISN3L4+xV+IpIfDTmKyISA93
tICISA/V8RURikL/Rq/AVkRip5ysiEgON+YqIxEA9XxGRGORv9Cp8RSRGesJNRCQGeZy9Cl8RiU8ijwc
eFL4iEhv1fEVEYlCgnq+ISN1Tz1dEJAYKXxGRGOiCm9SqZcuWcfaZp/Hu7FkkEgnuuGskz06ayMgRd
7Ndi+1IJODqa66n7+H9mPzsJK68fChlZWU0bNiQ62+4kYMO7h33KeS91atX87PeB1K2Zg3l5eUM+
OXRXDnsj+vXX/Tb87h/1H18uXTF+rKH/z2W6675I4lEgj1/0oX77n8AgCaNi+jUeU8AdthhR8Y9+njd
nswmrKCWs9fMRgJHAovcvXMoaw6MAdoB84GB7r7UzBLAzUA/YCVwsrvPCNsMBq4Iu73W3UeF
8u7AfUAxMAG4wN2TVR0jU1sVvjlwyYUX0KdPXx4cM46ysjJWrlzJs5Mmct4FF3LhRZfQuAhWl0d1t
922BeMee4I2bdowe9YsjjriMD78++LN4T0Bo1KgRTz8zmSZNmrB27VoOOWh/uKNN6a
zfNny79WfM2cON/55OJNfeIlmzZqxaNGi9euKi4uZNm3GWhmzj7m9x
savl8GHA50CEtP4HagZwjSYcBeQBJ4w8zGhzC9HTgDeJUofPsPsCT2c4RpXyeV6LnPj666956aWpnHz
qEAAaNmxI06ZNq6zftVs32rRpA0DHTp1Ys2YNZVZxq+CA3nO2StUSiQRNmjQBYO3atasaxduxYSCSoqKvjD
0Eu59n9v+F79u+++mzPP/g3NmjUDoGXLlnXe5s1RQSKR1VIdd58KLNmguD8wKnweBQxQxIKR/t7kl3
fxVoamatgcOASe6+JATuJKBvWLe1u7/i7kmigB9QzTaiMeQUeu3VjbPPO
I1vv/0WgDtuu4W9u/2ExOGsrJ+g3kkcfeZguXbvRqFGjum62pFFRUHPvbqxU9vtOeSQQ+nRoy
d33HYLRxx5FK1bt/5e3Q8/+hCOXcSsqjq3Q8++IC5H3xvJbgenEBr/eEHIgz9YTzzJpg0v/QK
2S0baXt3XwAQflb+ydgW+DSlXmkoy1ZeGlN5aRPtt9FHNmcQXeFrH5k7AhqVHqhGL8pTQs0akdWbbLL1m+jw/g6oS+AbmqhfrV1lX2b7x5nLJLzfSPdv0BpPsrtjEjhpHdiqEQPNZgGXoRx1a1yvpE44TppPDmsKRo6OYrbTGnHXt6K5yjmCdc5Ngq
sHevG//D3dxSQ2/AR6ibmoHGj6o7+ocNmk8ke+uf4+CZK75/FcuTYUL8Nnf0FDQ2RY5U1bSTQxCvUb
5e3Q8++IC5H3xA7wP//D9/odnJv5n/brVq1ezFv98jFv5nYqK9QRvw1bQ0twNs+LP21rUvm0hrNNvsv7nCfYa
Rx7RK74p5dQKG/cZVy5fzCjGtHWO4eD4zCUDbNUa2jzgEoXKLgLagAj5jdAb2ESqGQl8CDaOz7B39ZlVx
Dgs/d4XoiNbLNOuNHn+7ex7Zr6nkDPgH8wU0M1mB9VaphMqYqpSJj5o3kt7tAk0EXEbyEyH6ba3zm57
d4pAlDBw8qoH8Qb0Lg/HDq/iPsjoYrR9SPz4+4Y4j19

oqfEUkNhrzFRGJgcZ8RURioPAVEYmBhh1ERGKgnq+ISAzyOHsVviISozxOX4WviMRGY74iIjHQmK+I
SAzyOHsVviISoxykr5nNB1YAFUC5u+9lZs2BMUA7YD4w0N2XmlkCuBnoB6wETnb3GWE/g4Erwm
6vdfdRobw7cB9QDEwALnD3ZLbtzNkLNEVEqpPI8p8sHOzuXd19r/B9KDDZ3TsAk8N3gMOBDmE5
A7gdIIT1MKAn0AMYZmbNwja3h7qV2/XdmHNX+IpIbAoS2S0/Qn9gVPg8ChiQUj7a3ZPu/irQ1Mxa
A4cBk9x9x9ibsvBSYBfcO6rd39ldDbHZ2yr6wofEUkPoksl5pJAs+Y2RtmdkYo297dFwCEny1DeVvg05R
tS0NZpvLSNOVZ05iviMQmR7ea7evun5tZS2CSmb2fsQk/lNyI8qyp5ysisUkksltqwt0/Dz8XAY8Sjdk
uDEMGhJ+LQvVSYIeUzUuAz6spL0lTnjWFr4jEprZHHcxsSzPbqvIz0AeYBYwHBodqg4HHw+fxwCAzS
5hZL2B5GJaYCPQxs2bhQlsfYGJYt8LMeoU7JQal7CsrGnGnYQkfjU/qjD9sCjZgZRvv2fu//HzKYBY81sCP
AJcGyoP4HoNrO5RLeanQLg7kvM7BpgWqj3J3dfEj6fzXe3mj0dlqwmNGq7IidXlGzd2srlpXASry+
NuRd3YlP77yqXiBglWrc2bc621yPz4qzVZ/dJ22rZRvXkuQz1fEYmNHi8WEYlBHmevwldE4qOer4hIL
PI3fRW+IhKbH/nI8GZN4SsisdGwg4hIDPQmCxGROORv9ip8RSQ+eZy9Cl8RiY/GfEVEYqAxXxXGROO
Rv9ip8RSQ+eZy9Cl8RiY/GfEVEYlCQx+mrN1mIiMRAPV8RiU0ed3wVviISH91qJiISA/V8RURikMfZq/
AVkRjlcfoqfEUkNhrzFRGJgcZ8RURikMfZq/AVkfgk8rjrq/AVkdjkcfaSSCaTcbdBRCTvaG4HEZEYKHxF
RGKg8BURiYHCV0QkBgpfEZEYKHxFRGKg8BURiYEesqhDZtYXuBkoBO5x9+ExN0l+JDMbCbDwCRwJLHL3
znG3RzYf6vnWETMrRrBG4FDgc6Ar8ys47xtkpqwX1A37gbIZsfhW/d6QHMdfcP3b0MeAjoH3Ob5Ed
y96nAkrjbIZsfhW/daQt8mvK9NJSJSB5S+NaddFOIaGINkTyl8K07pcAOKd9LgM9jaouIxEx3O9SdaU
AHM2sPfAYcD5wQb5NEJC7q+dYRdy8HzgUmAu8BY919drytkh/LzB4EXom6pcwwB3hT0Hy+
IiIxUM9XRCQGCl8RkRgofEVEYqDwFRGGJgcJXRCQGCCt/NmJnNN7P3zewtM5tlZsfX4n47h88TzGyXa
uoPMLMeG3msk81sXHXtqGYfSTNrkuVx25nZ4my2WalNCt/N3zHu3gU4CbjXzFpsWCVwMC9HXNAdXe/HsDM9gduA1
YBr5IyD4WZzQeOdPdZZtYW+AfQIax+EJgB/Bw41MxOA/7m7m1qPNbDwG6L/7u7m1nD1nI0D
iJ70e78m52dmF4dzKgJWh/3NTKlyiZn1AbYFLnf3h8N2PYHHwNah3lXu/lRNjimSSwrfesLMDgYaA3
OAtsB+QJfKXquZnZFS9z/As+5+jpk1BAqqFBNqcoVeKyUvMFf3x8n0M+W7M+W7j7YjMbD0Kx391t
C+f7AQOAAd19jZgOzbDoW+AXYTTOB9kBnoEE4/vvE2rqIqs/c/d3ckdtiarzbYjWEpYjEWD2vDtqFPP1X
6ufsCM2sNTKvJUIZvIRil8N3/jzjNsWsBKoizqZAd1KwBgpbtPAaRAVsAD3sWZ214Y
HCWOq+wA/qyxz96rGTI8CugCvheMlgGZh3cHAKHdfC6w1s38R/UFRne5mdjnQHFgH7LbB+hGhT
W5mM4iCuZwo6J9OOe8ksCug8V6JlcJ383eMu89KU/5FNyucCotDZO4TeembWJQdtSgAj3f2qKtZlJf
TUxxH1pGeYWRuilYtMx0+Gn2+7+wFp9tku23aI1CZdcMsD7r4CeBEYWllmZjjuYWSuiMddiMzsglB8
DbJNmH98ALwMXpuyj8uLe1xts8wQwyMxKQr1CM+se1k0GTjKzbn/mzR1Tkb/mzR1T
gnH6gB0BV4L7e0QhmQq27y3mWX9B4BIbVP45o8TgY5m9o6ZvQOMAZq6+xrgV8CtZvYwuXbssYQaJ1TcG69MuivsfzbwJPBCdQ1396+Bq4GxY7Tp1JpzTUJv/mnz7
X7+DWwb7it7S2gcgav+4ETzGymmQ0Kr9b5b5AzA+1JvFd69MuivsfzbwJPBCdQ1396+Bq4jGa6cC36a
ptsbM/hv2eaa7L3L3pUQXA4eF2/HeA65rfIrVNs5qJiMRAPV8RkRgofEVEYqDwFRGGJgcJXRCQG
Cl8RkRgofEVEYqDwFRGJwf8DD56N48htMrEAAAAASUVORK5CYII=\n",
      "text/plain": [
       "<matplotlib.figure.Figure at 0x7f65cee69f28>"
      ]
     },
     "metadata": {},
     "output_type": "display_data"
    },
    {
     "name": "stdout",
     "output_type": "stream",
     "text": [

"2019-10-05 22:00:12.123777\n",
"total time all features xgboost and RandomOverSampler:481.5778818130493\n"
]
},
{
"data": {
"image/png":
"iVBORw0KGgoAAAANSUhEUgAAAYoAAAEWCAYAAAB42tAoAAAABHNCSVQICAgIfAhkiAAAAAl
wSFlzAAALEgAACxIB0t1+/AAAADl0RVh0U29mdHdhcmUAbWF0cGxvdGxpYiB2ZXJzaW9uIDIuMS
4yLCBodHRwOi8vbWF0cGxvdGxpYi5vcmcvNQv5yAAAIABJREFUeJzs3Xl4U2X2wPFvlu4LZakom6D6D6D
iGQUVFQERRAEVEHBHcRt3HcUNUVFQcV8BQdx+6qijo+MyjgKCIIoKKoKoCC4vAiIUWQoU6N4mu
b8/bgqldkmhyU3S83keHprkJvkfkNs2573mX67IsC6WUUqombqcDUEopFd00USillKqVJgqlfFKqVVJgqllFK1
lFKqVpoolFJK1UoThVJKqVppolAhE5ELGS203FEExxEpEJEDHNhvexGRQb6X2Hg4j8JCIn7MHz9
DMZAS6dRxGbRGQ10BLwAwXXXAR8AlY0yBg2E1KBgE1AHpCTwAHAMEgC+A240xv4PzzFyO0v4
OBB4ETgQTgD+AVYBLQFvgdSDDG+ClRT01ExAI6GmNMWhHk/7YmS99zYaIsitg0xxqQDXYAjgTscjm
ePVHdWLCLHArOBD4BWQAdgCfCfBlXmOaOcAxYGMBt6XY+892o6KGJUsEVei0RREHc2Y0zFzwJ5
qp62KGJUsEVxhTFmTvD2Y0Any4H/AzcaY4H/AzcaAucB1xpiPRKQJMAEY
hH0W/zJwjzHGLyKXBPfZS0SeAwqMMMaMqxfDQB8LkxZoKItAKeAo7HbvFMNMZDm43DugMlABD
gZFVz9JFZB6w1BhzbZX7ZwK5xpiLg6WK14FngJHB/Ywxxvy7rmNQ6blPATcDHwM3AK8B3QEv8CV
wjTEmR0QeBEYD5YAPeNUYc5YAPeMUYM6Ly2bSIvAIUAu2D7/tn4HxjzMpgPCcH97cv8G+gE/BadS0UEXkdaFr
x+6zm8fbYZ9eXAPcDqcFj/GDw8W7YLY9DgGLgv8HjXBZ83AJWZg0uyxs
wLbu8BbgcuB/YBlgOnB99Hb6AIsIDLjTFvichg7NZe++BxuMYY82PwtVYDzwGXAKkkASSsIfp6DsT8DH
ByM/d/GmJEisga7JVUUYPAwnBZ9/hTGmV/C1OwFPAkcHf1eJ7eLCMVei0RREHRKQNMBD7j63
Co9h/aF2Ag4DWwN3B7bsB/wJuBuKbvYX4UHYrSZSTgSuq2e0bwLkLki4gq+ZtPgbv8RETc
wDbsF0BroB9wkIqdUpwLvB/f/+7yvAq7Au0CO7n78D/iYjUdQwqPbcZsD9w
Ffbfw8vB2+2wvB+2wv6SmABhjxGdzsMt76caYEdWTo0R8i2vXXD/g1yqP7dsrrytMa
aw0u0/sM8m6zoGYJewSipuBFsyE4EB2C0CgAwR8Rhj/LXEW9mGSJ8swX
6ve7S/YEf4BOw+jVTsv/XFVZ672+9ARG7BTgitsEB/9K/G5ULsK9fgsXNsCM1YJ8FbTOOzNPnK+9pBOx1
XAf8KuI/I6dDkeHsN/6xKjqQRNFHDDGfB6+4o7q2HTf+ocrDfjvMAi9adAqbmeajBLbbfFsa
5BHsEsYZlV7nd2NMfF/Bnnnxx+yS5/yGbsSGXaxrPgU1rf9k3lfndmvajf/22
GbBCRLsD32Amm1phDsZ7Amp1fd55GE+Cve08++0xrJR18f6zO8+6/Gb3yWb1WyFK1h5Gg2:
+1tKVEVn2NMx1qeW2PnmDGmUES+xu68rXpGP4zdywlNRSStUrJoByyj7mNQXQy3YJ9Bdzf
GbBCRLsD32Amm1phDsB5oU3Ej2OprU/PmzAHOwi6F7YlnsWMfbozJF5GbsFt9e18PyLSG7sPoh9
2yycgInnseu8Vn5lllex7LfBgRX9JDWr7/f8GDA+WMM8E3hWXiJ9p9J+h4cQn6onTRTx40kM
XY8wPwVLPRBEZYYzYYzZJCKtGct7EmFnFnAS9hf8NOxv4j3AzKMMMb8Gx6Px+9SaFl6Gs7sDuHOwwBtjDGfV92hM
eZ7EckFgRmGWhALgR0icjcJ2MnSmiveicciv2F+WXuwv8mQAq8fAS6kRoRmNwkvFEmyicH9f7qUbC3Nm+wtN
Edu/xwT/CLrrZjUJM7OSyTUSaESzlVbIRu/N/T3wITBGR04HpwwDXYfSQ1uQdYJCKPA+ODiesgYBx2

J3RdMoAdQIGI/A34B/aghdq29wW38YrIaOwWRYUXgftF5GfsvpfDgHXGmC3sOi4VfWQvAP8TkTn
Yn4VU4ATgC2NMfl2Bi8iF2J+n3EqtUn8wtkBwX8ureep0YEIwKT6L3Yo5tFIJTO0h7cyOE8aYXOwO6
ruCd92O/Ye7QER2YJ+hSnDbhcCl2GWW7dh14/2Dz7sY+w/sZ+wS0LvUXgJ5E7vj9Y1KsfiBIdgdo79j
n92/iD2aJtT3Mx84BfuMcj12SelIoFfwjLPChmCcf2J3il9jjKkoV9V4DGrwJJASjHcBdqdoZZOAs0UkL9
jHELJgrf0c4DHsstKhwLfYLbjqtl+JnRTbAz+JyHbs/pNvgTq/bLH7P84PbvsC8FYd288CZmJ/Af+BXd+v
XB6agN2pPBs7Ab2EfazATl6visg2ERlmjPkWu59iCvbvZgX26KxQDcB+zwXYx/w8Y0yJMaYIe3DAl8F
99aj8pGASOgn7s7cBe+TWifXYr6qBDo9VMatiiKsxprYSTlQKllVygAuMMfXtMFcqorT0pFSEBIcHf4
Nd3roVu/4f6ogepRwTtkQhIv/ErhdvMsZ0ruZxF3azchD2aI1LjDHfhSsepaLAsdgluorS3ukVEyCVimZ
hKz2JSMWM3H/VkCgGAddjJ4ru2BNsuoclGKWUUnssbJ3ZxpgvgK21bHIadhKxjDELgCwRCWXcuFJ
KqQhyso+iNbuPqsgJ3lfrbFTLsiyXy1XbJkopFdcCAdi4EVavrv7fH39AaaXxdFfxPM9bV+/xF6eTiaK6o
Ousg7lcLnJzQxkdGP+yszP0WATpsdhFj8UusXosLAs2bXKxdq2LtWvdrF3rZs2aip9d5OS4KSmp/nu/
RYsAh/+tlMusl/il16W03t/D/vudu1fxOJkocrCn3Fdogz0WXiml4pplQW7urkSwZo27UlKw/68pETRv
HuBvfwvQrl2Atm0t2rbd9XObNgGaLF9Mxk3X4f3lZwqGFVJ86bXsvnJN/TmZKKYCI4LrBXUHthtjQlk
ETSmlopplwebNtSeC4uLqE0GzZgFEak4E6enVPg2Kikh79EFSnn8aVyBA8cWXUXLeBQ3yfsI5PPZN7
Gn7LYKLn92DveAcxpjnsBelG4Q9a7MIe6awUkpFPcuCLVsqJ4JdJaKK+4qKqk8ETZtadOwYCCYAK5g
EdiWFGhNBLRIWfEXGDf/As/p3/O07kD9xCuXH9d7Ld7lL2BKFMabWxbmMMRZwXbj2r5RSe8qyY
OvWmhPBmjU1J4KsLIsDD6w5EWQ06PUJba6tW3Gv+YOi626k8NY7IDW1QV9fZ2YrpRody4K8PKo
pC+1KBIWF1SeCJk0sDjig5kSQmVnt0xpc4uyZlB91DFaLFpQNGszWrxYTOKC6RaH3niYKpVTcsSzYtg
3WroUlS7zVjh4qKKg+EWRkWLRvXzURWMFkEKBJyEtbhodr82bSx95G8nvvUnL2ueQ/8wJA2JIEaK
JQSsWo7dthzZq/dhTbt93k51ckgpTdnpeebn/5VySAqp3GTieCGlkWSe+9Q/qY23Bv3Ur50V0pumF
kRHatiUIpFZV27IA//ti9g7hyX8GOHdW3CNLSKhKBxcEHe2nRomS3ElGTJhBrc3bd6/8kfdSNJH08Cy
s1lYL7H6b4imvAs3fDXkOliUIp5YgdO9h59l9dIti+vfpv89RUi/33D9Cjx65yUOVEkJW1KxHYE+7KI/iu
wsNVUEDi53Mp630C+eMnEWjfIaL710ShlAqL/Hx2KwtV7TTetq3mRNCuXYBu3apPBE2bxl6LYE94
Vq2AklL8h3bC3/Fg8j6ai79TZ0fevCYKpdQeKSioPRHk5dWcCNq2DdC1a3WJwKJZM6tRJIIa+XykPP8
MaY8+gL/DgeTN+QISEvB3PsyxkDRRKKWqVVBAlbLQ7p3GW7dWv/h0SoqdAI46yto5bLRy53Hz5o
08EdTC89MyMm6+joQfvifQIpvCW24Dr/Nf085HoJRyRGEhfxkpVDkRbNlSfSJITrYTQJcuvmoTQYs
WmgjqrbSU1ImPkzp5Ai6fj5JzzqPg/oexmjV3OjJAE4VScauoCHJy7A7i6hLB5s3VJ4KkJDsRHH64b2d
ZqPJ6Q9nZmggamquslOS33iDQcl8KnniSsn4nOx3SbjRRKBWjiourJgI7CaxfD6tWpdWYCBITLdq2tej
cefdEUPFzdraFO2yXNFM7FRbi/fVnfEcfg5WRyfbX3iKw//5YGRGa2l0PmiiUilLFxbBunauaSWV2csj
NrSkRQJs20KmT7y+zivffXxNBNEj44jMyRt6Aa/s28uYvJNByX0c7q+uiiUIph5SU2Ingr5PK7J83bar+2
zwhwaJNG4tDDqmaCOz5BZ06pbNlS2GE340KhWv7NtLuvYuU11/F8ngovu5GApnROhV8F00USoV
JaWntiWDjxuoTgddrJ4LevatPBPvsY9U6IVdbC9EpceaHpN92M56NG/B1Ooz8J6fgO+JIp8MKiSYKp
fZQRSKo3FFc+ecNG2pOBK1b24mg8qqjFX0F++5beyJQMciySPm/Z3DnbaXwzrspuu5GSEhwOqqQ
aaJQqgZlZTUlArtlsGGDC8v66/Afj8dOBL16+XbrH6hIBPvtp4mgUbAsvEu+x9flKHC5yJ84BVdZGf6D
xenI6k0ThWq0ysvtRFC5HLT76KHaE0HPnv4aE0EUzJFSDnLnrCX91ptI/HQO26bPxndM94ivz9SQ9
OOs4lZ5Ofz5567F5qrOJVi/3kUg8NdE4HbbieDYY/1UvWZx27YBWrXSRKBqEAiQ/MpLpN1/D+7CA
sr6nEig5b5OR7XX9OOuYpbPtysR5OXBTz8l7pYI/vyz5kTQqpVF9+41J4lYKh+rKOFZ+RvpN19P4oKv
CDTJYsfkZyk99/y4WMFQE4WKWj4frF9fc4vgzz9d+P2V/wiTAHC57ETQrVvNiSAx0Zn3pOJX8qsvk7j
gK0oHn0b+w09gtWzpdEgNRhOFcozfvysR/PHH7tcsXrvWzbp1VROBzeWy2G8/i65d/TvXGTr00CSy
sopo2zZA69aaCFRkeH5bjv/Ag8DtpvD2MZT37EXZgEFOh9XgNFGosPH7YcOG6hPBmjV2i8Dnq75Z
vu++AY4+elciqNxp3Lq1RVLS7ttnZyeRm+uPwLtSCigpIXXCY6Q+NZGCR8ZT8vfLIC0tLpMEaKJQe8H
vh40bK5aYcFF1Utm6dTUngpYtAxx5ZOAvZaF27apPBEpFC+/Cb8i4+Tq8vy3H36Yt/v3bOx1S2Gmi
UDUKBHYlgspDRyv6Ctatc1FeXn0i2GefAF26BHZelaxqIkhOjvCbUWpvFRSQ9tC9pLz0fwAUX34Vh
WPuwUrPcDiw8NNE0YgFArBpk2tna6C6RFBWVn0iyM4OcPjhNSeClJQxmlwixp9kxSX3we30Edy

Z8wBV+PY50OKWI0UcSxQAByc2tOBDk5NSeCFi0CdO68eyKoKBG1bh0gNTXCb0YpB7i25YHHg5W
RSekZZ5NfXEzJWcNobE1iTRQxzLLs0lDlfoFdfQV2IigtrTkRdOpUfSJo00YTgVKJ0z4gY/QtlA4aQsHjE8
HlouSCi50OyxGaKKLcli0uVq+uLhG4yMmBkpL0ap/XvHmAQw75a0dxRSJIS4vwG1EqRrg2biTjjlEkT
f8AKykJf9t29llZHEyc21OaKKLYp596GD48pdr1hpo1C9CpE+y3X/lfLlXZpk2A9Orzh1KqJpZF0ltvkH7
3Hbi3baO8+7HkT5yC/6COTkfmOE0UUezNNxOwLBeXXFKGyO6dxunpkJ2dQW5uidNhKhUXPCtXk
HHzCKzkFPIffoKSS6/Qi3sEaaKIUkVF8PHHXjp0CPDoo6WNudWrVPgEArjy8rCaN8d/UEfyJ06h/LjeB
Nq2czqyqKKJIkp9+qmXoiIXQ4aUaZJQKgw8vy0n4+YRYFlsm/oReDyUnneB02FFJW1XRanp0+0cPn
Soz+FIlIoz5eWkPvkETU/sScLCBfhbtcZVXOR0VFFNWxRRqLgYZs3y0q5dgMMOCzgdjlJxw7t0Cek3X
kfCsh/x79OSgscmUjZosNNhRT1tUUShzz7zUljoYujQci07KdVQSkvJHH42Cct+pPiCi8mbv1CTRIjC2q
IQkQHAJMADvGiMeaTK4+2AV4Gs4DajjTEzwhlTLJg61f61DBmiZSel9pYrfwdWRiYkJVHwxCSslBTK
+5zodFgxJWwtChHxAE8DA4FDgeEicmiVzcYCbxtjjgTOA54JVzyxorR0V9mpSxctOym1p1wF+TBiBE
2PO8ZeigMoGzBIk8QeCGfpqRuwwhizyhhTBvwHOK3KNhaQGfy5CfBnGOOJCZ995qGgwMXgwT4
tOym1hxI+/Zimx/eAp5/GyszEnZvrdEgxLZylp9bA2kq3c4DuVbYZB8wWkeuBNKB/KC+cnR2/y/p+/L
H9/8UXJ5KdXfdl2uL5WNSXHotdGu2x2LIFRo6Ef/0LvF646y68Y8bQTC9wslfCmSiqOx+2qtweDrxijB
kvIscCr4lIZ2NMrTWX3Nz8hooxqpSWvvvvp9O6tUWHDoXUdRJkz8yOz2NRX3osdmnMxyLz/PNJ
mjOb8iOOJH/iFJqd2DN4LMqcDs1xe3PyEM7SUw7QttLtNvy1tHQ58DaAMeZrIBloEcaYotq8eR527
NCyk1L1Uly888fCO++h4K772DbzE/ydD3MwqPgSzkSxCOgoIh1EJBG7s3pqlW3WAP0AROQQ7ETR
aIuJU6cmADB0aLnDkSgVAyyL5Ddeo/nRnfD8tAwAf+fDKL7+JrvspBpM2BKFMcYHjABmAb9gj276S
UTuE5Ghwc1uAa4UkSXAm8Alxpiq5alGoawMZs70st9+AY4+Wkc7KVUb9x+raXLO6WTcdB2UlOJZ
/bvTIcW1sKbd4JyIGVXuu7vSzz8Dx4Uzhlgxf76H7dtdnHtuuS5YqVRN/H5SXnqetIfuw1VURGn/kyl4
/EkCrds4HVlc0/ZZlKiYZDd4sE6yU6omqZMnkPbw/QSaNSP/iUmUnjWsUV9QKFI0UUSB8nKYOTOB
li0DdOvmdzocpaKLzwceD7hcFF96Be516yi8fQxWdrbTkTUaWuSIAvPne8jLs0c7adlJqV28P3xH0/7
Hk/T+fwGwsppS8MSTmiQiTL+WooAuKa5UFcXFpN17F1kD+uL9eRneZUudjqhR09KTw3w+mDHD
S3a2lp2UAkj4aj7pN4/A+/sq/Pu3J3/CU5T37uN0WI2aJgqHffWVhy1b3Fx6aRkej9PRKOWshHmfk3
XWECy3m6JrRlA4eiykpjodVqOnicJhuqS4UkAgAG435T17UTz8QkouvhTf0cc4HZUK0kThIL/fLju1aB
Hg2GO17KQaH9eWLaSPvZ1A6zYUjh0HHg8Fkxr91QaijnZmO+jrrz1s3uxm0CCflp1U42JZJP3vXZr1
6kryf98m4av5doedikqaKBw0bZqOdlKNj3v9n2T+fTiZV1+Gq6iIgnsfYtu0Wbo+UxQL6TcjIunAAcaY
H8McT6Ph99vDYps3D9Czp5adVOPg2ryZpsf3wL19G2W9jid//GQCHQ5wOixVhzoTRfC61y8AfqC9i
BwD3GWMGVr7M1VtFi70kJvr5qKLyvRESsU/ywKXC6tFC0ouuBj/gQdRcuHfdfmNGBFK6ek+7CvT5
QEYYxYBB4YzqMZARzupRsHvJ+XZKWRedpGdLIDCcQ9QctElmiRiSEh9FMaYqhcc0stF7YVAwC47N
W1qcdxxWnZS8cnzy89kndqf9HvuJGHBl7hz1tb9JBWVQkkUhSKSTfAypiLSG9ge1qji3MKFHjZudD
NoUDkJCU5Ho1QDKysj9fGHadq/NwnfLabkzHPYOv9bAm3bOR2Z2kOhVMfvxL74UAcRmQMcCp
wW1qjiXMXaTlp2UnHHssg641QSFn2Df79WFDw+kbKTBzodldpLdSYKY8zXItIP6AW4gC+NMVvCHl
mcCgTsYbFZWRa9e2vZScUZl4uSYcPxHdKJwrvvxcps4nREqgGEMuppvDHmFmBaNfepelq82M369
W7OO0/LTio+JMz/gtQnx7P91TcgLY2Sv1/mdEiqgYXSR3FiNff1behAGoupU+3sMHRoucORKLV3X
Du2k37LDWSdOZiE+Z+TOP8Lp0NSYVJji0JEzgLOBvYXkTcqPdQEKA53YPGoYrRTZqbF8cdr2UnFrsR
ZM0m/9SY8G9bjO6QT+ZOextflKKfDUmFSW+lpFfAJ0DP4f4UdwMfhDCpeff+9m3Xr3AwbVk5iotP
RKLVn0h68l9RJ47ESEii8fQxF19+MfqDjW42JwhjzPfC9iHxgjMmNYExxq6LsNGSIlp1U7CrtfwoJX80
nf/xk/H87xOlwVASEMjx2q4hcBnQBkivuNMZcFbao4pBl2WWn9HSLE07QspOKHe51OaTffScFd91
LoH0HfN17sG36bJ1Z3YiE0pn9HHbn9RnAWuxhsvpNV08//OBm7Vo3p5ziIynJ6WiUCkEgQPIrL9G0
d3eSpr1Pyuuv7npMk0SjEkqi6AFcBOQZY+4HjgPahzOoeKRLiqtY4lm1giZnnErGbTeD203+k09TOO
Yep8NSDgklURQbYyzALyIpxpg8oHWY44orlmX3T6SlWZxwgiYKFd0SZ35I0xN6kvj1l5QOHEze/IWU
nH+RtiIasVD7KJoAs4HpIrIZ0M7teli61M2aNW7OPLOclBSno1Gqdr6jjsbfpi2Fo8dSNuR0TRAqpEQx
FCjHXvPpIiALeCWMMcWdiiXFBw/W1oSKQqWlpE58nPJu3SnvexKBlvuSN38RuPUCmMoWylpPFU

uK+wkmCBEZCMwMX1jxw7Jg2rQEUlMt+vXTRKGii/fbhWTcPAKv+ZXy7seyre9J9gOaJFQltSYKETk
DaAfMMMb8JiL9gQeBpmiiCMmyZW5+/93Naadp2UlFkcJC0h65n5T/exaXZVF82ZUUjh3ndFQqSt
W2hMdE7OXEvwOuFpH/Af8AxgHPRiS6OFCxpLiOdlLRwr1qJVnDzsCzZjW+Aw+iYOIUynv0dDosFc
Vqa1EMBI4wxuSLyL7AaqCLMebXiEQWBypGO6WkWPTtq4lCRYdAm7YEMjMpvWEkhaNGQ3Jy3
U9SjVptiaLIGJMPYIzZICLLNUnUzy+/uFm50s2QIeWkpTkdjWrMEmdMx71xAyWXXgGJiWybNRdd5
16FqrZE0UJEKi/T0aTybWPM/4UvrPhQMdpJy07KKa5Nm0i/81aSp/6PQEYmpWcPw8rI1CSh6qW2
RPE50LvS7S8q3baAOhOFiAwAJgEe4EVjzCPVbDMMu9/DApYYY84PKfIYMH26l+RkHe2kHGBZJL3z
H9LvGo07L4/yrt3If/JpO0koVU+1rR570d68sIh4gKeBk4AcYJGITDXG/Fxpm47AHcBxxpg8Edlnb/YZ
TX791c3y5R5OPbWc9HSno1GNSkkJmeefTdInH2OlppH/0GOUXHoleDxOR6ZiVCgT7vZUN2CFMW
YVgIj8B3sU1c+VtrkSeDq4LAjGmE1hjCeiKtZ2GjJEWxMqwpKTsTIyKOtzIvnjJxNot7/TEakYF85E0Rp
7tdkKOUD3KtscDCAiX2KXp8YZYz6q64WzszMaKsawmTEDkpLg/PNTyAhjuLFwLCKlUR8LY+Ddd2H
MGACS//0apKTQXJffaNyfiwYSzkRR3SfUqmb/HYETgDbAPBHpbIzZVtsL5+bmN0iA4bJ8uZuffkpjwI
BySkpKKCkJz36yszOi/lhESqM9FuXlpDz7FGmPP4yrtJS8o3rQdGA/cgv9UFjgdHSOa7Sfi2rsTcIMeZ6
+iDSt52vnAG0r3W4D/FnNNh8YY8qNMb8DBjtxxDRdUlxFgnfpErIG9CX9gXFYmU3Y/tJr+Lp2czosFY
fqTBQi0lVEfgd+rHQ7lJnZi4COItJBRBKB84CpVbZ5Hzgx+LotsEtRq+oRf1SaOtVLYqLFySdrolDhkfrEI2
SdfAIJS5dQct4FbJ2/kLIhpzkdlopTobQonsReQXYzgDHmW+D4up5kjPEBI4BZwC/A28aYn0TkPhEZ
GtxsFrBFRH4G5gK3mO21P9tRI8VK1z88ouHE0/0k6kjEVWYWImJBFq1Zttb/yN/8rNYTZs5HZKKY
6H0USQZY5aKSOX7ymrauDJjzAxgRpX77q70swWMDP6LC9Om2ROZBg8udzgSFVcKCkh94VmKrrs
REhMp/sf1FF92FTr2WkVCKImiTERSCXZEi8jfCDFRNEbTpnlJSLAYMEDLTqphJMz9hIxRN+JZuwaG
W8ceAAAgAElEQVQrLY3iq661Z1br7GoVIaEkioeAj4FWIvIicCpwSTiDilWrVrlYtsxD//4+mjRxOhoV6
1x5W0m/ZwzJ//k3lsdD4c2jKL74MqfDUo1QKBcu+lBElgMDsIe8PmaMWR72yGLQ9On2Gd7QoVp
2Unsn8ZPZZNxwLe7cTZQf3oX8iVPwH3a402GpRqrORCEiw4H3jDFPRSCemDZ1qhev1+1+KUU7TspP
aeK38HBWPvpfja68EbzilPStUulFFP5wJrRORZEak6s1oFrV7t4scfPRx/vJ+m9Z1xopRlkfSff+PesB6As
n4ns2XRUopvuFmThHJcnYnCGHM60Al7MtxzIvKziNwa9shiTMVoJ13bSdWXe80fNBl2Opk3/IO0c
WN33m+1bOlgVErtEtLMbGPMZmPMk9iT4+YBf1kuvLGbPt2Lx2DKD2T6gQBQIkv/gczY7vQeLn
cynr25/CMfc4HZVSfxFKH4ULOAW4FDtRfAj0DXNcMWXNGhfff++hTx8fzXTekwqBe9VKMq+/hoR
F3xBo2pT8xyZQes55oIv4qSgUSvEzB1gOvApcZowpwpDG9IsWf6dF3bSdWTy4V32Y+UDD2Dgocex9o
nbi7FouJQKIniOGPM6nAHEsumTUvA47EYNEgThaqZ98cfIBDA1+UoAh0OYOvnCwi07+B0WErVqc
ZEISI9jDELgINF5OCqjxtjZoc1shiRk+Ni8WIPvXv7aN686irqSgHFxaQ98Qgpz0zGf8CB5H2+ALxeTRIq
ZtTWorgKWADcVc1jFqCJAi07qdolLPiK9JtH4F25An+7/Sl46HEd7qpiTm3XzL4s+H/vyIUTe6ZNS8Dt
1rKTqqKggPT77ybl5RexXC6Krr6WwtF3QVqa05EpVW+hXI/i81Dua4z+/NPFokUeevb0k52tZSdVict
F4qdz8B0sbJs+m8L7H9EkoWJWKG3g3a6fJyJuIDs84cSWDz+0D9/gwdqaUODaugXvsqWUH38CpK
Wx/a338Ldua188XakYVltn9i3AKKCZiFS+hGka8G64A4sFU6d6cbksTj1VE0WjZlkkTf0f6XeMgpJS8r
5cRGC/VvgPOMjpyJRqELW1KP4JfABMAa6rdP8OY0xuWKOKARs2uFi40EOfn5attSyU2Pl3rCe9N
tGkvTRh1jJyRTePpZAts6JUPGlts7sPCAPe3lxVcWHH3qxLJeOdmqsLIvkN14j7Z4xuHdsp6xnL/InPEX
ggAOdjkypBldb6ekVY8wlIvI1wavbVWaM6RnWyKKclp1U0rT3IRAg//EnKbnoEnCHtHSaUjGnttLTM
8H/x9ayTaO0caOLBQs8dOvmZ999tezUaPj9JHw5z+6sdrnIn/AUWBaB1m2cjkypsKqt9LQw+P8nFf
eJiBfIMsZsjkBsUaui7KRLijceHvMrGTddR8LiRWz734eUH9ebQKvWToelVESEMo/idRFpIiIpwDJgpYj
cFP7QolfFbGwdFtsIlJWROv5RmvbrRcLiRZSceTY+TY+OcTpqJSKqFCKqp2NMduBQcAXQCug0V7hPTfX
xVdfeeja1U+rVmvlp2imfeH76j6cknkPbogwSat2D7a2+R/9w/sVq0cDo0pSIqlERRUZ46HvgwuMx4IH
whRbcZM7wEAi6GDtULFMW7pOlT8f68jOKLLiVv3jeUnTLQ6ZCUckQoM7ONiMzEvhzqncESVKM1
daqWneKZ97tv8R1xJHg8FN5yO2X9T6a8R6Me4KdUSC2Ki4CXgL7B1kRzYExYo4pSmzfbZaejj/bTpo
2WneKJK38H6bfeTNMBfUl56Xn7zpQUTRJKEUKiMMYUAZ8AB4rIyUChMebDsEcWhWbO9O/wO3ux
g8WMtO8STx449o2rs7Ka++hO9vh1B+9DFOh6RUVAll1FN/wAC3A3cAv4pIo7xm9rRpdtlJl8eJlh8XGB9

eWLWT84wqaXDAMd+4mCkeNJm/OPHyaKJTaTSh9FA9jl52WAYhIJ+zrZ3cNZ2DRZutWmDfPw5F
H+mnXTstO8SDhyy9I/u/blB95FPkTn8Z/aCenQ1IqKoXSR5FYkSQAjDE/EVqCiSsffVRRdtLWRCxzr/8
TV95WAMqGnM72f77OthmfaJJQqhahJIrNInJhxQ0RuQDYEr6QotPUqQkADBmi/RMxybJIfu0Vmv
bqRvo9wbEYLhdlg4eCx+NsbEpFuVBaBv8A3hCR57EXB/wFGB7WqKLMtm3wxRceDj/cT/v2WnaK
Ne5VK8kYdSOJ878gkJFJ+THdnQ5JqZhSZ6IwxiwHuopIVvD2trBHFWU++siLz6dLisccv5+U558h7dE
HcBUXU3rKQAoem0hgv1ZOR6ZUTKltmfE2wBOAAN8BtxljGl3JCWDaNLvspMNiY4tn1UrSHrgHKy
uL/EnPUHrameByOR2WUjGntj6KF4CNwN3B7Z6ISERRZvt2+OwzD507+zngAC07Rb2yMtzr7Sv3+j
sezI7/e4Wt8xZRevpZmiSU2kO1lZ7aGGMGAojIDGBRfV9cRAYAkwAP8KIx5pEatjsbeAc4xhjzbX33E
04ffeSlvFyXFI8F3u++JeOm67CSktk28xPweu3OaqXUXqmtRbGzzmKM8df3hUXEAzwNDAQOBYaL
yKHVbJcB3AB8U999RML06XbZSRcBjGKFhXDLLWQN6o/311/wHXkUlJU5HZVScaO2FsXBIvJVTbdD
uBRqN2CFMWYVgIj8BzgN+LnKdvcjGjQo46QnbsgLlzPRxyiJ8DD9SyUzRKmPc5GSOvhz9W4+9w
AAUTp1Des5fTYSkVV2pLFKft5Wu3BtZWup0D7DYuUUSOBNoaY6aLSMiJIjs7Yy9DC83s2faJ6fDhn
ojts76iNa6IKCmBEVfBxo1w2214x40jK6VRL268U6P+XFShx2Lv1XYp1E9qeixE1fUc7jwtFxE3MBG4
pL4vnJubv+dR1cPrrycDCfTtW0hubvRdgiM7OyNixyKauDZuxGrZEoCEyc9hNWlC05P62MeioPEdj6
oa6+eiOnosdtmbhBnKzOw9lQO0rXS7DfBnpdsZQGfgMxFZDfQApopIVKwhVVAAc+d6+dvf/Bx8cP
QlicbItXkzGVdfSrMTeuDabF+2vbzPifi6HOVwZErFt3Cu2bQI6CgiHYB1wHnA+RUPBi+vuvOakiLyGT
AqWkY9zZ7tpbRU13aKCpZF0n/fJn3s7bi3bqX86GNwFeTrJUmVipCwtSiMMT5gBDALe9mPt40xP4
nIfSIS9WMWK5YU19nYznKvyyHzwmFkXnslrpISCh54hG3TZxNo38Hp0JRqNFyWVfdoHhHpAxxijHl
ORPYBMowxK8MeXfWscNccCwrg0EPTads2wPz5RVE7T6sx1F+bnHcmiZ/Ooez4E8kfP4nA/u2r3a4
xHItQ6bHYRY/FLtnZGXv8TVZn6Sk4GukMYB/gOSAZeAXovac7jXaffOKlpMSeZBetSSKeubZvw2qSB
UDB/Y+QMPQbSoZfqDOrlXJIqNfMPgEoADDGrAGywhiT46ZO1SvZOcLnI2XKJJp1ORTvD98B9jIcJed
fpElCKQeFkiiKjTFVpyXH7TCgwkK7RXHggQEOPTRu32bU8SxbStbAfqTfdxekpODautXpkJRSQaGMe
soRkR6AJSIu7Gtn/xLesJzz6adeiopcDB1apiexkVBaSurEx0idPBGXz0fJsOEU3PcQVrPmTkemlAoKJV
HcALyOPeehCFiAPdQ1LlWMdtJhsZGROnkCaRMex9+6DQVPPElZv5OdDkkpVUUoFy76E+gbXLzPH
Zz/EJeKi+35Ex06BOjcWctOYVNUBCkp4HJRfM11uMrKKLpxJFa6LrWgVDQKZdTTyVVuA2CMmR2
mmBxTUXYaMkTLTuGS8NmnZIy6kcJRoyk97wKsjEwKx9zjdFhKqVqEUnq6q9LPycBhwA9A3CUKn
WQXPq5teaSNG0vKG69heTy4N25wOiSlVIhCKT3tNl9CRA4Drg9bRA4pKYFZs7y0axfgsMO07NSQE
j+cRvrtI/Fs2kh558MpeHIKvsO7OB2WUipE9V7CwxizFDgiDLE4au5cL4WFLoYOLdeyUwNK+HQOTS
69APf2bRSMuYdts+ZqklAqxtS3j8INHBPK82JNRdlJJ9k1AMsCnw8SEig/oS9FV19HycWX4u94sNOR
KaX2QH37KHzASmBYeMJxRmmpXXZq2zZAly5adtob7py1ZIy6EV/Hgym8/xFwuym8/2Gnw1JK7YV
aE0Xw4kIPGmM+ilA8jvjsMw/5+S4uvFDLTnssECD55RdJe2Ac7sICLJcL/H7weJyOTCm1l2rtozDGBIC
7IxSLY6ZNSwBg6NCqK5WoUHhW/EbWaQPJuGMUJHjZMflZdrzxriYJpeJEKJ3Z34nI0WGPxCGlpfD
RR15atw5w1FFadqov16ZNNO3fm4RvvqZ0yOlsnbeI0vMu0EX8lIojofRR9ACuFpFfCK4gC2CM6Rm
2qCJo3jwPO3a4GD5cy071EgiA2421zz4UXX8zPjmEssFRfz0qpdQeCCVR3B72KBw0dapddhoyRMt
OISkpIXXCYyR8v5jtb78PLhdFt8T1R0SpRq/GRCEiLxljLjfGfBLJgCKprAxmzvSy334BunbVslNdvN8sIO
Pm6/Cu+A1/23a4/1xHoHUbp8NSSoVZbX0UR0YsCofMn+9h+3YXgwf7cIft6uFxoKCAtDtvJWvoKXh
WrqDoymvY+vkCTRJKNRJxN3GuPnSSXQgsi6zTBpKwdAm+jgeTP2EKvu49nI5KKRVBtSWKw0RkUz
X3uwDLGLNPmGKKiPJymDEjgZYtA3Tr5nc6nOhjWfbIJZeL4quvpWzlbxTdfBskJzsdmVIqwmpLFMu
BQZEKJNK+/NJDXp6Lyy8v17JTFYnTPiD1qQls/+80rIxMSocNdzokpZSDaksUpcaYPyIWSYRp2emv3
Bs3kD56FEkfTsVKSsL77SLKT+zndFhKKYfVdi5dFrEoIszngxkzvGRnB+jeXctOWBZJb75O017dSPpwK
mU9epI39ytNEkopoJZEYYyJ2x7Lr77ysGWLm8GDfbrKBJA25jYyb7wWfD7yHxnP9vdn4D+oo9NhK
aWiRKMc9aRlp92VnnMenjV/UPDIeAJt2jodjlIqyjS6bly/Hz780EuLFgGOPbZxlp08yw1NzhyM57flA
PiOPJodr7+tSUIpVa1GlygWLPCwebObQYMaYdmpvJzUiY/TtO9xJM7/gqSp/3M6IqVUDGh0paepU
+23PHRo4yo7eZd8T8aN1+H9eRn+lvtS8OgEygYNdjospVQMaFSJoqLs1Lx5gJ49G0/ZKen9/5Lxjytw

+f0UX3AxheMewGqS5XRYSqkY0agSxcKFHjZtcnPRRWV4G9E7L+vVB98RXSi88x7Kjz/B6XCUUjGm
UfVRVJSdBg+O77KTqyCf9NtHkjhjOgBWixZsm/mpJgml1B5pNOfVgQBMn+6laVOLXr3it+yU+Mls0
kfdhGddDp6VK3f1Q+hVmZRSe6jRtCgWLvSwcaObgQPLSUhwOpqG59q6hYzrrqLJ8LNxb9xA4S23s
/3fbzsdllIqDoS1RSEiA4BJgAd40RjzSJXHRwJXAD4gF7gsXOtLTZ8ev6OdPMsNWacPwr05l/IuR5I/8
Wn8nTo7HZZSKk6ErUUhIh7gaWAgcCgwXEQOrbLZ90BXY8zhwLvAY+GIJRCwZ2M3aRKfZSf/AQfiP
/AgCu55gG0zPtEkoZRqUOFsUXQDVhhjVgGIyH+A04CfKzYwxsyttP0C4MJwJwBLJ4sZv1692cd145iYn
h2EOEWRbJb7wGvmL4+9Xg9bLtg5noeulKqXAIZ6JoDaytdDsH6F7L9pcDM0N54ezsjHoFMmeO/f+
FFyaQnR3jHRSrVsFVV8Enn0Dz5mRffTVk1O94xKv6fi7imR6LXfRY7L1wJorqhtlY1W0oIhcCXYE+obx
wbm5+yEFYFrz9dhqZmS6OOKKA3NyQnxpd/H5SXnyOtIfvx1VUROlJp5D0zxfJLQFKQj8e8So7O6Ne
n4t4psdiFz0Wu+xNwgxnosgBKq8y1wb4s+pGItIfGAP0McaUNnQQ333nZt06N+ecU05SUkO/eoQ
UF5N15mASFi8i0KwZ+eMnU3rmOWTvkwn6R6CUCrNwJopFQEcR6QCsA84Dzq+8gYCTwPDDD
GVHd97r02bZpdaho6tDwcLx8ZKSn4D+qIv107Ch58HKtFC6cjUko1ImHr/TTG+IARwCzgF+BtY8xPIn
KfiAwNbvY4kA68Iyl/iMjUhozBsuzRTunpFn36xNZoJ+/3i0m79y77TQD5E54i//mXNUkopSIurPMoj
DEzgBlV7ru70s/9w7n/JUvcrF3r5qyzyklODueeGlBREWmPPUTKc1NwBQKUnnYGvi5HEZezBJVSMS
Gux1PG2pLiCV/Oo+mJPUl9ZjKBdvuz7b3pdpJQSikHxe1aT3bZKYG0NIsTToj+RJF2952kPjcFy+2m6
NobKLztTkhNdTospZSK30SxdKmbP/5wc+aZ5aSkOB1N3QKtW+M75FDyJ07Bd1RXp8NRSqmd4rb
0NG1adC8p7tq8mbR7xkBJCQDFV1xD3sdfaJJQSkWduEwUlgVTpyaQmmrRr1+UJQrLIum9d2jW+x
hSn32K5Ndfse/3eIiP9UWUUvEmLhPFTz+5+f13Nyed5IuqspP7z3Vk4XnuqquoiIL7HqLk0iudD
ksppWoVl30UFWWnaBrtb/wCIeAu07OB2WUkrVKe4SRUXZKSXFom/f6EkUg
eYtwO0mf8JTlFxwsV5xTikVM+Ku9PTLL25WrnTv7+PtDQHA/H7SXluCu4cewHd8l7Hs/W7ZZRc+
HdNEkqpmBJ3LYqKSXZDhjjXmvD88jjRfy+2m6imD7dS3KyRf
/+DiSKsjJSn3yC1EnjcZWXU3L2uRQ88Ejdz1NKqSgWV4ni11/dLF/uYdCgctLTI7tvzy8/k3n1pXh//QV
/q9YUPPEkZf1PiWwQSikVBnHVR+HkaCcrIwP37h1s/k8sO0SShlIobcdWimDbNS1KSxcknRyZR
JMz7HCshEV+PYwm0acvWb37QZcVnEnbloUy5e7+fVXDyee6At72cm1fRvpI68n66whZNx8Hfjt
a11oklBKxaO4aVFUlJ3CPdopceaw6aPCPdopceaHpN92M56NG/Ad0on8SU/by28opVSciqtEkZwcOk
L/1JpLffw8rMZHC0WMpGnGTrs+klIp7cZEoVq508cMPboYN85Ka2rDv7f5zJd533yJw9GfvvzFTHGv7
W/h2ZFSSkWZuEgU06bZeTE/XvS4H79bfXfg+R12ZwkdMqS8tZ0EvjyUW/XvS4H79IfKRswCJKT2f7faQRa7qulJqVUoxIXndlTp3pJSLA
YMKCByk6BAMkvv0jTXt3IvOYy3H+us+9Vq0ThFKq0Yn5RLFqlYulSzKccXkGzZvWvWF9uBxfveSjNmB9vyCBoRR6ujpDnjPU2/NumLXz
m4fCV4v+Q8/QWC/Vnv/wkopx3z++Vx69erKH3+s3nnfd999y2233bTbdg8+OI4JE/gpz
jvvDO46KJhXHnlxXz99Zd7Hctrr73Mueee0Q1HCedNIDTz8bgPnzP+eppyYyYcJTCeTexVTKGI+UUyb5s
X3zuzvt//33VXscTipgvPU2fbpedhg7dy7JTIEDK05NJe/whXCUllA4aQsGj4+1kzvDD+vCD6f+/C
nDmzuPzyq0N6zgsvPMstBdb1/hh1e/D999/tVR76H76ZzE7wMHGrJNM6M5cXOfbzYuSR1yqXxnv9H9j2gn6kscWNQAy0VmJo9Bmj00K9B3YRlnPSFFCa7n6vzHmmfdmrRiT8BnSBFv0NVWTJ8jxqVxuNrQFdVUeQjtbKMbCa1Dwe7X35tEYBxGoJKD/9NnMrdrJmo4vA4Mz4k7eA3sHQlGuSqmDerfZqHN16bw+Xpr/k/
/ybWTO+Dt9PQ/Gm+7aNUQP8Lf1cKrUfJb/JjcjF83T8kmmVZ6qE4MQoRNn3sWS3WxCrruWjxB
k4vAMEcgM5Exr3sxbGpvOIspxB/5rxkXqOuzRRjbP7GQyfNoSXf/TQs/DJDI+G/DdOcVXSGs3uy
/Izzfxhd2zJHpq+zH/2JeZy7ofxkEchkihmJZA/DB+lDQS+yJN2r/Y+7ccJJfdBvNDmUbE7ivV+uYm69vM5/3/AetV
JYnBSgjVzU6Xg7P9LBJ36jDmx5LjS5IqEs1ppDffhJzzyHt3BuCtO+fnnlA9XYwOYE+9ox1CqOtjSKE
VRlPvt8T4bDwfYIhF+HcLsM1DrV94/9r/9vDQt0mJl7GTIM/HJqBjTN38jI/sT2Gx+HnjuW3UTjTHyOdT
GAf+h7aQ2Bv3iWbe3IHVs3/DwBOfV3LlHYhe2rUR7rfQwhfbzDhh/Z4f/7yJvuUWsRN2Uz+3C3ARN7NtM
rs53C9dwrTiy6d74CHkBZyQlsYqD2g2EwE5U71FQWpHEM3Tw1wu/gZcEqkzUY2eiAedgXuY4KE+A6
VPi+mZWb5HGdg7PkafI/QjcoBjc63d6fwQ+n1ADYRmnv3APYGzB53hZBTmzZkvjTYE6o3wFh6W+z17e
Av47rzQNtnVBoOgBUN4KqmqQ/P28QiY/qk85zqPIrfD7eTRWnz/BaHxt7Jbqq+x1FJvhjqFnyC0+e4O
6ILsuLbEqc1G6UdpxVzymh0pNOi/QYzaRMxTEkzNvtjY7hKbNuS5VGdDhmJWBhbLkOsObmRZXKvdF
dc5GPsYGzC8jV6HYJ0uqTTH9OtXjTpsm2Fe+N1/Q/IyB1NLrAn17/nFZuBC/vd8Ui/wyJR11SAJSBe7
fCeUCmGjz4Gr+P9+V2IJwu2hUq5C8VaZfS5OT9F/9B8A2RS14z8jd4Rf2p/h3q8jI1HxkfpvhYxPdiCQA

MbNqxnxYrf6NhRSE5OZvDgoTz55BPceuudJCQksHnzZhYvXsgppwzabR/1aVEcd9zx3HvvWM499
wI2b85l7dq1HHJIp922ycjIoLCwgDVr/qBdu/359tsF7L9/ewDWrl1D27btAPjqq/m0adNuD49M/cRs
otiTJcUTFi7AvS6HohtvofCW2yE5OVzhKaWiwIcffsiwYRfudl+fPn35+OOPOOKII7nrrvt46KF7KSsrw
+v1Mnr0WNKDq4peeeW1vPDCM1x44TkkJiaSnJzCFVdcs1fxHHDAgfTt258LLzwHj8fDyJG37RzxNG
rUDYwefRctWmRz221jGdxxx92A3T/x7bcL8Xq9ZGRkMGbMuL2KJ1Quq7qiWXSzc
nPzGTgwlR9+cLN0aSEtWtT8HhLmfkJ57z7g9UJZGZ7fluMPNuliXXZ2Brm5+U6HERX0WOyix2IXPRa
7ZGdn7PEQqZiccJeT42LxYg89e/prTBKuTZvIuOLvZJ17BinPBMcZJybGTZJQSqlIicnSU62jnSyLpLffJP
2u0bi3baP8mO6UDTw1whEqpVT8iMlEMW1aAm63xaBBuycK99o1ZIy6kcS5n2ClppH/8OOUXHol
uGOy4aSUUlEh5hJFTg4sWuThuON87LPP7mUn79IfSZz7CWUn9iP/iUkE2kZmRIBSSsWzmEsU771
n/19RdvKs+I1AVlOsFi0oGzSYSYbe9Np/y43rr8hlJKNZCYq8m88w64XBannlxMyqTxND2xJ+ljb9v5eH
mv4zVJKKVUAwpri0JEBgCTAA/wojHmkSqPJwH/Ao4GtgDnGmNW1/aa9Yd/LPZceSsHQJxcMvZO
uXiygbPLShw1dKKRUUztJTN2CFMWaVMaYM+A9wWpVtgRPtNeGtf78LtBPRGqtG41kAr59W7Ptrf9R
MOkZrKw9WTZWKaVUqMJZemoNrK10OwxtM2xhifiGwHmgOzuzDKDGruzsDKdDi
Bp6LHbRY7GLHou9F84WRXUtg6rTqEPZRimllIPCmShygLaVbrcB/qxpGxAk2ArWGMSSmlVD2Fs
/S0COgoIh2AdcB5wPlVtpkK/B34GjgbgB+NQYoy0KpZSKImFrURhjfMAMIYBbwC/C2MeYnElPRCqGKb
0ENBeRFcBIYHS44lFKKbVnYnGZcaWUUhEUczOzlVJKRZYmCqWUUrUwKWUUrWK2kUBw7H8R6wK4ViMBK4
AfEAucJkx5o+IBxoBdR2LStudDbwDHGOM+TaCIUZMKMdCRIYB47C47CHnS8xxlQdUBIXQvgbaYc9uTc
ruM1oY8yMiAcaZiLyT2AwsMkY05AUnNE8CBgFFwCXGmO/qet2oGbUXjorQE+i4K0bMokaRYoWkRIU86/bwmYM75ert
Rx7hvtjkY0yMkI8FohIBnAD8E1kI4ycTj6c8JE9OjUn2WiWCjSEcKG4gGFRo1qqJBihPb/8Z9iRTJvQL6HE6DVRNS5t2Np/s8VpMxwGnhdzKw8ePEEEgs5aW0Wq8n/zJ6w0DFw
mFWBALY8PBDoG/10FPBvKi0ZloiBMy3/EqDqPhTFmrjGmKHhzafaclXgUyucAjTS7DsP8DjvH7zRtiahSyeAiLJRjcS
XwtDEmD8AYsynCMUZKKMfCAjKDPzeg93wnC4j2oAm
d0zbBobgVy3/Em1CORWWXAzPDGpFz6jwWInIk0NYMZ2SgTkglM/FwcDBIvKliCwIlmfiUSjHYh
woYjkADOA6yMTWtSp7/cJEL2JQpf/2CXk9ykiFwJdgcfDGpFzaj0WIuLGLkPeErGInBPK58KLXWI4A
RgOvCgiWWGOywmhHIvhwCvGmdDbY9fnXgp+XxmaPvjej9UDp8h+7hHIsEJH+wBhgqDGnEKxR
VpdxyID6Ax8JiKrgR7AVBHpGqGkAIyjUv5EPjDHlxpjfAYOdOOJNKMficuBtBtAGPM10Ay0CIi0UWXkL5
PqorWUU+6/McudR6LYLnleWBAHNehoY5jYYzZTqU/fhH5DBgVp6OeqvkbeZ/gmbSItMAuRa2KaJ
SREcqxWAP0wz4Wh2AnityIRhkdpgIjROQ/2Kt5bzfGrK/rSVHZotDlP3Y8Dg8DqD74jIDyIy1aFww
yrEY9EohHgsZgFbRORnYC5wqzFmizMRh0+Ix+WQ4+Ix+IW4EoRWQK8iT0sNO5OLEXkTckTcuggFEAAAUBSURB
VOyTZxGRHBG5XESuEZFrgpvMwD5ZWAG8AFwbyuvqEh5KKaVqFZUtCqWUUtFDE4VSSqlaaJQSi
lVK00USimlaqWJQimlVK2idR6FaiSCE+NK2LUu01xjzM11PCcH6G+M+bUB9v8A9ppI64EkYD5wrTG
mfA9e6zrAY4yZLCJHAQcYYY94NPuBgusR7bXgcusGHHyoAE4HFjzMshPO9MYE2czi9RYaCJQkW
Ds40xyxzc/8vGmNEikgx8gZ046r26qDHm6Uo3jwL6wHHYTHGD3RpgFirOsMY86uIHAEsEpxpiNd
TznTOyEqlIChUQThYpKInR9iSqROy1aEYaYz6r6rZv7gGFAKeAH+hhj8kXkWOBh7ImIAHcZY2pdLNE
YUyIi8wEvvapwAPY1y/ypwAPY1y/YCFxtjFkVnNn7MpDPBB2F5wBnNxCmcrusfPBlsnXiBCCdQlQaI/EBwshtQHnzOcGCQMeac4
H4SsBdqOwZ7iYU7gNOxWwlrgCvrmnFvjFkiIvlAK2CvMIYk2CjiHQBBpgCp2C2l54wxT4nI1jvoEJ2E9boz5t4
hcBlwTjD8PuMYY81tt+1SNh/ZRqGjwbnBG+Q8ickrwvvhnGmO7B6wdciH2Rqt2ISDZwI9DFE
AcoFpFm2C2Cc40xbXbGXVn5BRDKrvkaV18sSCTgK+F5F9+27iVExziK4/h3XMplgVDChuJX5JJbLhOa
LMXSWGBsbFiwkVKuibBQSpLrCA1JNNRETEQRkt2xYKGkKCURYSzOefUY7zzzzzm5qzmc17zP/3v/f/3v/f/3v/f/3v/f/3v/f/3v/f/3v/f/3v/f
xfPv3P+T+d4GfvVhT4f52PoJryG0oxoDnOu+D3xUN8DtJnZzCqptCtAg6QR8Xk58NLM3uJlacYD82P
td6ihyKOkJXjNnkpk9hpoMLNZeM2rTZImR7OeW8C+uLcLkpbiG1N9jD8CnOxuztR3ZESReoNqqadJ
UY5gLN65b5ykUWb2sTDmE/5APC+pDWiNaKIemAC0SaqM7QAmAi+qzL8+SnB3ANfwTWkl8LRw
DnIKOCppCJ6e2i9pMB4ttPdksWb2RVIrHlkcA5rwCAVgZ6ieh73PgDv4NiVa3H+MGDGNiVa3H+MREvCFk5WxkK
HJc0DfgNjAGmA6+qfMcKPFX2JOaswwPG17I7S8z+ylpLlAPNOCRwDL8

```
QffczBpqnOuMmf1TKyyaYFWtb2NmLZIe4tHHdjwKaKp5Ze4scEDSFWAhsCqu1wG7zOy/CKoLlTO
K1cBFSZPM7ANwAE9brTGzX5Lu0un3K6gDTpjZnh6uIfURmXpKvdUw4E38vQHP1/8jUkmjzKzdzHb
gBeGmAg+BKZIWF8bO6+H8j4A5kibH5/XAEzP7Gi1G38UbRnvxDmudfY41dKUdGA3sA66aWeWtr
xvAxkrfCEmDJE3v7mbN7BIe3WyNS8OBt7FJzAAWldzbDWCdpLExZ39Js7ubM/UdGVGk3moz0Bq
vgN7DOxh2NgK4HOmgOvwtnutm9l3SSuBgPHAH4imq5bVObmbvJTUBLdHg5gOwNv7dCDRK+oF
HHdV6Ud8GtkS10rv4YXbx+zskNQM7gQWF62ckjQTuRxqoH3AUeFnDbW8DHks6hJ+RNEtah1cKf
VAY1wycltQIHI5zit3AzVjrQDyie1bDnKkPyOqxKaWUSmXqKaWUUqncKFJKKZXKjSKllFKp3ChSSimV
yo0ipZRSqdwoUkoplcqNIqWUUqk/Jx0d3Dg1QXoAAAAASUVORK5CYII=\n",
     "text/plain": [
      "<matplotlib.figure.Figure at 0x7f65cc3c1160>"
     ]
    },
    "metadata": {},
    "output_type": "display_data"
   }
  ],
  "source": [
   "\n",
   "data = requests.execute(output_options=bq.QueryOutput.dataframe()).result()\n",
   "from sklearn.utils import shuffle\n",
   "data = shuffle(data)\n",
   "\n",
   "from sklearn.preprocessing import StandardScaler\n",
   "train_size = int(len(data) * .8)\n",
   "\n",
   "train_icdmed = data['diag_med'][:train_size]\n",
   "train_label = data['label'][:train_size]\n",
   "\n",
   "test_icdmed = data['diag_med'][train_size:]\n",
   "test_label = data['label'][train_size:]\n",
   "\n",
   "vocab_size = 800\n",
   "tokenize = kr.preprocessing.text.Tokenizer(num_words=vocab_size,\n",
   "                                      filters='!\"#$%&()*+,-./:;<=>?@[\\\\]^_`{|}~\\t\\n',\n",
   "                                      lower=True,\n",
   "                                      split=\",\",char_level=False)\n",
   "tokenize.fit_on_texts(train_icdmed)\n",
   "x_train = tokenize.texts_to_matrix(train_icdmed)\n",
   "x_test = tokenize.texts_to_matrix(test_icdmed)\n",
   "\n",
   "train_sex = data['sex'][:train_size]\n",
   "test_sex = data['sex'][train_size:]\n",
   "vocab_size = 2\n",
   "tokenize = kr.preprocessing.text.Tokenizer(num_words=vocab_size,\n",
```

```
"                                   filters='!\"#$%&()*+,-./:;<=>?@[\\\\]^_`{|}~\\t\\n',\n",
"                                   lower=True,\n",
"                                   split=\",\",char_level=False)\n",
"tokenize.fit_on_texts(train_sex)\n",
"x_train_sex = tokenize.texts_to_matrix(train_sex)\n",
"x_test_sex = tokenize.texts_to_matrix(test_sex)\n",
"\n",
"x_train=np.hstack((x_train,x_train_sex))\n",
"x_test=np.hstack((x_test,x_test_sex))\n",
"\n",
"scaler = StandardScaler()\n",
"age=data['age']\n",
"age=scaler.fit_transform(age.reshape(-1,1))\n",
"age_train=age[:train_size]\n",
"age_test=age[train_size:]\n",
"x_train=np.hstack((x_train,age_train))\n",
"x_test=np.hstack((x_test,age_test))\n",
"\n",
"\n",
"from sklearn import preprocessing\n",
"encoder = preprocessing.LabelBinarizer()\n",
"encoder.fit(train_label)\n",
"y_train = encoder.transform(train_label)\n",
"y_test = encoder.transform(test_label)\n",
"\n",
"from imblearn.over_sampling import SMOTE\n",
"from imblearn.combine import SMOTEENN\n",
"from imblearn.combine import SMOTETomek\n",
"from imblearn.over_sampling import RandomOverSampler \n",
"from imblearn.over_sampling import ADASYN \n",
"from sklearn import preprocessing\n",
"from imblearn.under_sampling import ClusterCentroids\n",
"from imblearn.under_sampling import RandomUnderSampler\n",
"\n",
"\n",
"print(\"Before OverSampling, counts of label '1': {}\".format(sum(y_train==1)))\n",
"print(\"Before OverSampling, counts of label '0': {} \\n\".format(sum(y_train==0)))\n",
"\n",
"sm = RandomUnderSampler(random_state=42)\n",
"X_train_res, y_train_res = sm.fit_sample(x_train, y_train)\n",
"\n",
"t0 = time.time()\n",
"\n",
"\n",
```

```
    "from xgboost import XGBClassifier\n",
    "import time\n",
    "t0 = time.time()\n",
    "gbm = XGBClassifier(max_depth=4, n_estimators=20000, learning_rate=0.05,
nthread=60).fit(X_train_res, y_train_res.ravel())\n",
    "y_pre = gbm.predict(x_test)\n",
    "\n",
    "t1 = time.time()\n",
    "total = t1-t0\n",
    "\n",
    "cnf_matrix = confusion_matrix(y_test, y_pre)\n",
    "from sklearn.metrics import f1_score\n",
    "print(\"f-score metric in the testing dataset: {}%\".format(f1_score(y_test, y_pre,
average='binary') ))\n",
    "from sklearn import metrics\n",
    "fpr, tpr, thresholds = metrics.roc_curve(y_test, y_pre)\n",
    "print(\"AUC of test dataset is: {}\" .format(metrics.auc(fpr, tpr)))\n",
    "class_names = [0,1]\n",
    "plt.figure()\n",
    "plot_confusion_matrix(cnf_matrix , classes=class_names, title='Confusion matrix')\n",
    "plt.show()\n",
    "print(datetime.datetime.now())\n",
    "print(\"total time all features xgboost and RandomOverSampler:{}\" .format(total))\n",
    "\n",
    "fpr, tpr, threshold = metrics.roc_curve(y_test, y_pre)\n",
    "roc_auc = metrics.auc(fpr, tpr)\n",
    "\n",
    "# method I: plt\n",
    "import matplotlib.pyplot as plt\n",
    "plt.title('Receiver Operating Characteristic')\n",
    "plt.plot(fpr, tpr, 'b', label = 'AUC = %0.2f' % roc_auc)\n",
    "plt.legend(loc = 'lower right')\n",
    "plt.plot([0, 1], [0, 1],'r--')\n",
    "plt.xlim([0, 1])\n",
    "plt.ylim([0, 1])\n",
    "plt.ylabel('True Positive Rate')\n",
    "plt.xlabel('False Positive Rate')\n",
    "plt.show()"
   ]
  },
  {
   "cell_type": "code",
   "execution_count": null,
   "metadata": {},
```

"outputs": [],
"source": []
},
{
"cell_type": "code",
"execution_count": null,
"metadata": {},
"outputs": [],
"source": []
},
{
"cell_type": "code",
"execution_count": 1,
"metadata": {},
"outputs": [
{
"name": "stdout",
"output_type": "stream",
"text": [
"Collecting xgboost\n",
" Using cached
https://files.pythonhosted.org/packages/c1/24/5fe7237b2eca13ee0cfb100bec8c23f4e69ce9df
852a64b0493d49dae4e0/xgboost-0.90-py2.py3-none-manylinux1_x86_64.whl\n",
"Requirement already satisfied, skipping upgrade: scipy in
/usr/local/envs/py3env/lib/python3.5/site-packages (from xgboost) (1.0.0)\n",
"Requirement already satisfied, skipping upgrade: numpy in
/usr/local/envs/py3env/lib/python3.5/site-packages (from xgboost) (1.14.0)\n",
"Installing collected packages: xgboost\n",
" Found existing installation: xgboost 0.6a2\n",
"   Uninstalling xgboost-0.6a2:\n",
"   Successfully uninstalled xgboost-0.6a2\n",
"Successfully installed xgboost-0.90\n"
]
}
],
"source": [
"!pip install xgboost -U"
]
},
{
"cell_type": "markdown",
"metadata": {},
"source": []
}

],
"metadata": {
 "kernelspec": {
  "display_name": "Python 3",
  "language": "python",
  "name": "python3"
 },
 "language_info": {
  "codemirror_mode": {
   "name": "ipython",
   "version": 3
  },
  "file_extension": ".py",
  "mimetype": "text/x-python",
  "name": "python",
  "nbconvert_exporter": "python",
  "pygments_lexer": "ipython3",
  "version": "3.5.6"
 }
},
"nbformat": 4,
"nbformat_minor": 2
}
